# Deep Learning-Based Classification of GAD67-Positive Neurons Without the Immunosignal

**DOI:** 10.3389/fnana.2021.643067

**Published:** 2021-03-31

**Authors:** Kotaro Yamashiro, Jiayan Liu, Nobuyoshi Matsumoto, Yuji Ikegaya

**Affiliations:** ^1^Graduate School of Pharmaceutical Sciences, The University of Tokyo, Tokyo, Japan; ^2^Institute for AI and Beyond, The University of Tokyo, Tokyo, Japan; ^3^Center for Information and Neural Networks, National Institute of Information and Communications Technology, Osaka, Japan

**Keywords:** fully convolutional network, somatosensory cortex, motor cortex, mouse, GAD67, NeuN, deep learning, interneuron

## Abstract

Excitatory neurons and GABAergic interneurons constitute neural circuits and play important roles in information processing. In certain brain regions, such as the neocortex and the hippocampus, there are fewer interneurons than excitatory neurons. Interneurons have been quantified via immunohistochemistry, for example, for GAD67, an isoform of glutamic acid decarboxylase. Additionally, the expression level of other proteins varies among cell types. For example, NeuN, a commonly used marker protein for postmitotic neurons, is expressed differently across brain regions and cell classes. Thus, we asked whether GAD67-immunopositive neurons can be detected using the immunofluorescence signals of NeuN and the fluorescence signals of Nissl substances. To address this question, we stained neurons in layers 2/3 of the primary somatosensory cortex (S1) and the primary motor cortex (M1) of mice and manually labeled the neurons as either cell type using GAD67 immunosignals. We then sought to detect GAD67-positive neurons without GAD67 immunosignals using a custom-made deep learning-based algorithm. Using this deep learning-based model, we succeeded in the binary classification of the neurons using Nissl and NeuN signals without referring to the GAD67 signals. Furthermore, we confirmed that our deep learning-based method surpassed classic machine-learning methods in terms of binary classification performance. Combined with the visualization of the hidden layer of our deep learning algorithm, our model provides a new platform for identifying unbiased criteria for cell-type classification.

## Introduction

Neural circuits consist of glutamatergic excitatory neurons and GABAergic interneurons (Tremblay et al., 2016). While excitatory neurons, or principal cells, transmit information to downstream neurons, interneurons gate signal propagation by the relevant inhibition and sculpt cortical network dynamics. Particularly in the neocortex, interneurons constitute a minority of the neuronal population (Meyer et al., 2011), but they substantially contribute to information processing in the cortex, such as gain control of cortical circuits (Isaacson and Scanziani, 2011; Katzner et al., 2011; Bryson et al., 2020; Ferguson and Cardin, 2020), sensory feature selectivity (Sillito, 1975; Tsumoto et al., 1979), response reliability (Kara et al., 2000; Movshon, 2000), and temporally precise regulation of excitatory neuron firing (Cardin, 2018). Consistent with this notion, the loss or malfunction of interneurons is associated with neural and psychiatric diseases, such as epilepsy, bipolar disorder, and schizophrenia (Benes and Berretta, 2001; Marín, 2012; Goldberg and Coulter, 2013; Lewis, 2014). Moreover, neuroscientists have characterized neurons from morphological, electrophysiological, and neurochemical perspectives (Mihaljević et al., 2019); however, these criteria often vary between researchers, which prevents unbiased classification and hinders a better understanding.

To reliably identify interneurons and quantify their loss, GAD67, an isoform of glutamic acid decarboxylase, has been widely utilized because it is highly immunoreactive in the cell bodies of interneurons (Esclapez et al., 1994; Shetty and Turner, 2001; Meyer et al., 2011). While GAD67 is a reliable marker protein for GABAergic neurons (Ribak et al., 1978; Ribak, 1978; Staiger et al., 2002; Meyer et al., 2011), the expression levels of other proteins also vary between excitatory and inhibitory neurons. For example, NeuN, a neuronal-specific nuclear protein (Mullen et al., 1992; Kim et al., 2009), is widely used as a marker protein for postmitotic neurons and is differentially expressed among cell types in the murine cortex, hippocampus, and cerebellum (Weyer and Schilling, 2003; Yu et al., 2015). Thus, we questioned whether the fluorescence patterns of immunostained NeuN and counterstained Nissl allow for the classification between GAD67-positive and GAD67-negative cortical neurons.

To address this question, we implemented deep learning-based methods to analyze fluorescence pattern data. Deep learning is a subclass of machine learning algorithms based on a multilayered artificial neural network that extracts high-dimensional feature patterns (i.e., output) from the given raw dataset (i.e., input) and has recently benefitted image processing. For example, the lower (e.g., the first and the second) layers extract geometric features such as edges and their arrangement in images, whereas the higher (e.g., the penultimate and the last) layers identify abstract concepts and complicated characteristics. Deep learning-based image processing methods have recently improved and been applied in biological fields (He et al., 2015; Jiménez and Racoceanu, 2019; Kusumoto and Yuasa, 2019; Liu et al., 2020).

We performed immunohistochemical staining of neurons in the primary somatosensory cortex (S1) and the primary motor cortex (M1) of mice and manually annotated these neurons as GAD67-positive or GAD67-negative. We then attempted to classify them using any combination of immunofluorescence signals with the aid of a custom-made deep learning-based algorithm (Mihaljević et al., 2015; Chen et al., 2016; Xiao et al., 2018). We further compared the currently established algorithm against a conventional machine learning-based algorithm to evaluate the classification performance (Li et al., 2006; Kang et al., 2017).

## Materials and Methods

### Data Acquisition

#### Animal Ethics

Animal experiments were performed with the approval of the animal experiment ethics committee at the University of Tokyo (approval number: P24–10) and following the University of Tokyo guidelines for the care and use of laboratory animals. The experimental protocols were followed as per the Fundamental Guidelines for the Proper Conduct of Animal Experiments and Related Activities in Academic Research Institutions (Ministry of Education, Culture, Sports, Science and Technology, Notice No. 71 of 2006), the Standards for Breeding and Housing of and Pain Alleviation for Experimental Animals (Ministry of the Environment, Notice No. 88 of 2006) and the Guidelines on the Method of Animal Disposal (Prime Minister’s Office, Notice No. 40 of 1995).

#### Histology

Three young adults (8-week-old) male ICR mice were anesthetized with 2–3% isoflurane gas. Anesthesia was confirmed via the lack of reflex responses to tail and toe pinches. The mice were transcardially perfused with chilled phosphate-buffered saline (PBS) followed by 4% paraformaldehyde in PBS, and then their brains were removed. These brains were postfixed in 4% paraformaldehyde overnight and washed with PBS three times for 10 min each, and coronal sections were prepared using a vibratome at a thickness of 100 μm. The anterior-to-posterior coordinates of the sectioned slices were estimated based on the published literature (Franklin and Paxinos, 2019) so that the sections could include the S1 and the M1. For each mouse, we collected 12 sectioned slices from 0.7 mm to 1.9 mm anterior to bregma. The sections were blocked with 10% goat serum (GS) and 0.3% Triton X-100 in PBS for 60 min at room temperature. These sections were incubated with rabbit primary antibody against neuronal nuclei (NeuN; 1:500, ab177487, Abcam, Cambridge, UK) and mouse primary antibody against glutamate decarboxylase 67 (GAD67; 1:500, MAB5406, Merck, NJ, USA) in 10% GS and 0.3% Triton X-100 in PBS for 16 h at room temperature unless otherwise specified (see also Supplementary Figure 1). The sections were washed three times for 10 min each with PBS and incubated with Alexa Fluor 488-conjugated goat secondary antibody against rabbit IgG (1:500, A11034, Thermo Fisher Scientific, MA, USA), Alexa Fluor 594-conjugated goat secondary antibody against mouse IgG (1:500, A11032, Thermo Fisher Scientific, MA, USA), and NeuroTrace 435/455 blue fluorescent Nissl stain (1:500, N21479, Thermo Fisher Scientific, MA, USA) in 10% GS and 0.3% Triton X-100 in PBS for 6 h at room temperature.

#### Confocal Imaging

Before we captured images of each set of slices, we adjusted the laser power for each fluorescence channel to the maximum just below the intensity that would cause fluorescence saturation. The images were captured with the following laser configurations: channel 1 for Nissl (excitation wavelength, 405 nm; HV (PMT voltage), 700 V; laser power, 0.7–5.0%; gain, ×1.0; offset, 0%), channel 2 for NeuN or GABA (excitation wavelength, 488 nm; HV, 700 V; laser power, 0.6–0.8%; gain, ×1.0; offset, 0%), and channel 3 for GAD67 (excitation wavelength, 543 nm; HV, 700 V; laser power, 12.0–47.0%; gain, ×1.0; offset, 0%). The images (1024 × 1024 pixels, 12 bits/pixel, 16-bit intensity) for each region of interest were acquired at a sampling speed of 2.0 μs/pixel at Z-intervals of 0.5 μm using a laser scanning confocal microscope (FV1000, Olympus, Tokyo, Japan) equipped with a 20× or 40× objective lens (20×, UPLSAPO20X (NA, 0.75), Olympus; 40×, UPLSAPO40X (NA, 0.90), Olympus). We captured 10 slices (i.e., approximately 5 μm along the Z-axis) for each region of interest (ROI) primarily in layers 2/3 of S1 and M1.

### Data Analysis

The images were processed and analyzed using ImageJ software (National Institutes of Health, MD, USA) and Python 3. The summarized data are presented as the mean ± the standard deviation (SD) unless otherwise specified. *P* < 0.05 was considered statistically significant. The original *P* values were adjusted with Bonferroni correction and compared with 0.05 when multiple pairwise comparisons were required.

### Preprocessing (Erosion-Dilation Algorithm and Manual Annotation)

In our deep learning scheme, single-cell images in S1 and M1 that were extracted from the captured images of the sectioned slices were used as training data. We extracted the first and the last (10th) in the stacked images, split the color channels into three separate channels (e.g., GAD67, NeuN, and Nissl), and applied U-Net, a pre-trained deep neural network (Falk et al., 2019), to the images of the NeuN channel to specify the cell positions. We obtained binary images of the cells, created the ROIs, and excluded particles smaller than 180 pixels^2^ from the following analyses (Figure 2A).

**Figure 1:**
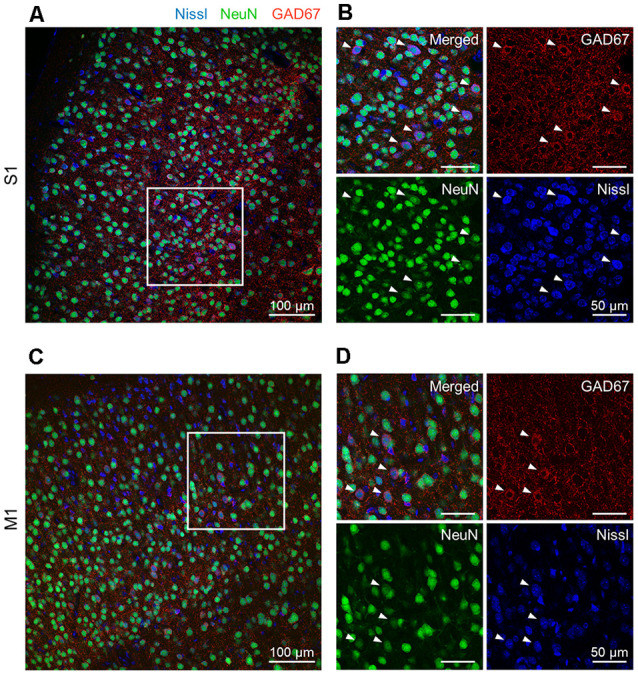
Representative photographs of GAD67 and NeuN immunoreactivity in the S1 and the M1 of mice. **(A)** Representative image (1,024 × 1,024 pixels, 16-bit intensity, 20×) of a section of S1 immunostained with GAD67 (red) and NeuN (green) and counterstained with Nissl (blue). **(B)** Magnified image of the boxed area (white). High-magnification images of merged (top, left), anti-GAD67 (top, right), anti-NeuN (bottom, left), and Nissl (bottom, right). White arrowheads point to GAD67-positive cells. **(C)** The same as **(A)**, but for M1. **(D)** The same as **(B)**, but for M1. Abbreviations: S1, primary somatosensory cortex; M1, primary motor cortex.

**Figure 2:**
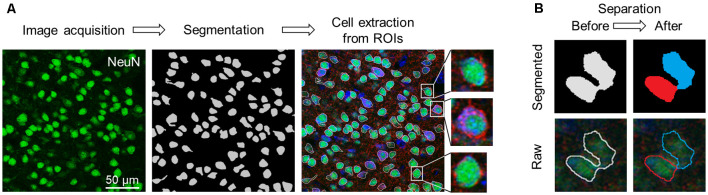
Flowchart of image acquisition and preprocessing. **(A)** Workflow of cell extraction using the pre-trained segmentation network. **(B)** Diagram of the erosion-dilation algorithm. ROIs that enclosed multiple cells (left; pale gray) were separated into two or more parts (right; blue and red). Top: Segmented images. Bottom: Raw images. Abbreviation: ROI, region of interest.

When we captured the images, we adjusted the laser power so that the fluorescence would be barely under the saturation level for each Z-plane and kept the image acquisition conditions equal across all the Z-planes. For each channel, we then calculated the mean and the SD of all (i.e., 1,024 × 1,024) the intensities and linearly normalized the original pixel intensities in each Z-plane image between the mean + the SD and the mean − the SD.

Since the neurons in the neocortex are tightly packed, multiple cells were considered as a single ROI (Figure 2B, *left*). For a given misestimated ROI, we first eroded the contour of the ROI to separate it into multiple plots, which were subsequently regarded as new ROIs. We then dilated them back to their original sizes using custom-written Python scripts, and the separated ROIs were discarded if they were smaller than 180 pixels^2^ in size. Note that this erosion-dilation algorithm affected only the size of the ROIs and that the pixels of the original image were never modified. These processes allowed almost every ROI to contain a single cell (Figure 2B, *right*). From the original (1024 × 1024 pixels) image, rectangular cell images were cropped out to tightly enclose the corresponding ROI and manually annotated by three independent skilled professionals with the aid of images with three channels (i.e., GAD67, NeuN, and Nissl). Annotations by any given pair of two professionals were significantly related for S1 (*χ*^2^ = 1211.26, *P* = 6.54 × 10^–265^, *df* = 1 (experimenter 1 *vs*. experimenter 2); *χ*^2^ = 1385.32, *P* = 9.74 × 10^–303^, *df* = 1 (experimenter 1 *vs*. experimenter 3); *χ*^2^ = 641.59, *P* = 4.52 × 10^–141^, *df* = 1 (experimenter 2 *vs*. experimenter 3)) and M1 (*χ*^2^ = 1211.25, *P* = 6.54 × 10^–265^, *df* = 1 (experimenter 1 *vs*. experimenter 2); *χ*^2^ = 1218.93, *P* = 4.69 × 10^–267^, *df* = 1 (experimenter 1 *vs*. experimenter 3); *χ*^2^ = 560.22, *P* = 7.54 × 10^–124^, *df* = 1 (experimenter 2 *vs*. experimenter 3)).

### Neural Network Architecture

Neural networks, such as a convolutional neural network and a recurrent neural network, typically consist of two primary steps: forward propagation and backpropagation.

### Forward Propagation

In forward propagation, the input data are put through and manipulated by various layers (e.g., convolutional, recurrent, pooling, and dropout layers). Here, our FCN model contains: (i) convolutional, (ii) dropout, (iii) batch normalization, and (iv) pooling layers (Figure 4).

(i) In convolutional layers, the input is transformed into a feature matrix of reduced size through convolution by kernel matrices. This processing with kernels enables the extraction of meaningful features from the input into a smaller number of parameters (Khan et al., 2018).
(ii) During the learning processes of deep neural networks, we often observe phenomena called overfitting. Overfitting occurs when the model learns unnecessary details from training data to the extent that the model exerts negative effects on predictions on new data. To prevent this problem, we implemented dropout layers. During the learning process, a dropout layer ignores some of the layer outputs, which break undesirable co-adaptations among artificial neurons, making the model more robust (Srivastava et al., 2014).
(iii) The batch normalization layers are applied to standardize the layer inputs. Without these normalization layers, the distribution of every input would be different, which negatively affects the learning process of a machine learning model. Implementing the batch normalization layers increases the learning efficiency and the learning speed of the model (Ioffe and Szegedy, 2015).
(iv) The pooling layer generally scans the output of the network by calculating a summary statistic of the neighbor outputs. This step helps decrease the number of computations by reducing the size of the output features and removes unnecessary information from the feature matrices. Our model implements global max pooling, which extracts the maximum value of the feature matrices and converts it into a vector. The output vector is then passed on to the final layer, where the probability of input classes is calculated using the Softmax function (Hinton et al., 2015).

**Figure 3:**
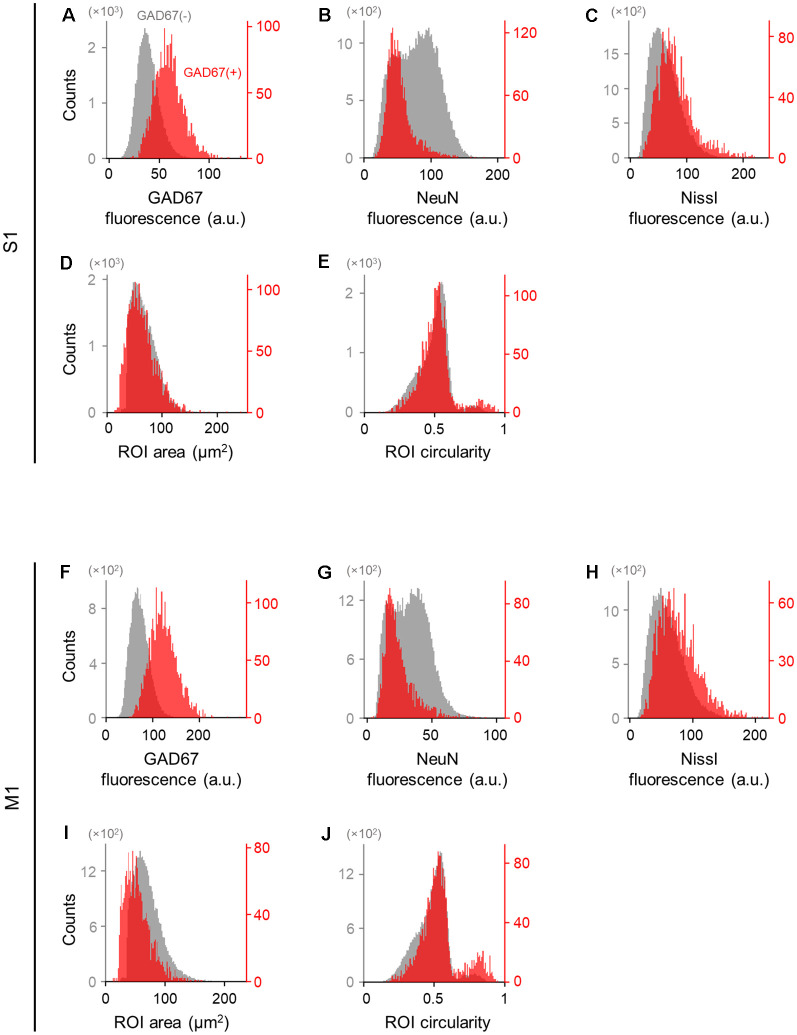
Differences in the immunofluorescence and shape of GAD67-positive and GAD67-negative cells in the primary somatosensory and motor cortices. **(A)** Histogram of the fluorescence of anti-GAD67 signals of manually annotated GAD67-positive (red) and -negative (gray) neurons in S1. **(B)** The same as **(A)**, but for anti-NeuN immunofluorescence signals. **(C)** The same as **(A)**, but for Nissl fluorescence signals. **(D)** The same as **(A)**, but for the area of individual neurons (i.e., ROIs). **(E)** The same as **(A)**, but for the circularity of individual neurons (i.e., ROIs). **(F–J)** The same as **(A–E)**, respectively, but for M1. Abbreviations: S1, primary somatosensory cortex; M1, primary motor cortex; ROI, region of interest.

**Figure 4:**
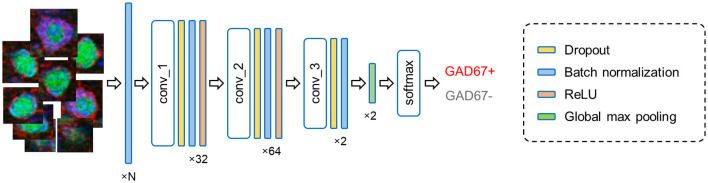
Fully convolutional network architecture. The current model processes input cell images (leftmost) in multiple layers and outputs the results of the binary classification (rightmost). The numbers (bottom) imply the channel features in each layer; note that N represents the number of fluorescence channels and thus ranges from 1 to 3. Abbreviation: conv, convolutional layer.

### Backpropagation

The feedforward operation is performed by manipulating the weights in various layers in the model. Initially, the weights of the model are randomly generated. These weights are updated in every training iteration by a process called backpropagation.

During every training epoch, the training data are separated into smaller datasets called batches. The output of the batches is compared with their true labels. The comparison of the output and the true labels yields a metric called loss, which is used to calculate an average gradient of the output layers. This gradient is then backpropagated to update the network weights.

### Deep Learning-Based Algorithm

After the preprocessing, we implemented a fully convolutional network (FCN), a deep learning-based method, to classify the cell images. First, since our dataset of images of GAD67-positive and GAD67-negative cells was imbalanced, we downsampled the GAD67-negative cell images to a ratio of 1:1. The downsampled class was upweighted by the downsampling factor to calibrate the model and ensure that the outputs could be interpreted as probabilities.

Our FCN was implemented using Keras, a Python deep learning library, and the TensorFlow backend. The network was optimized by adaptive moment estimation (Adam) or stochastic gradient descent (SGD) with learning rates of 0.001 or 0.0001, respectively. The parameters for the optimizer Adam were as follows: *beta*_1_ (i.e., an exponential decay rate for the 1st moment estimates) = 0.9, *beta*_2_ (i.e., an exponential decay rate for the 2nd moment estimates) = 0.999, and *epsilon* = 1e-7, whereas the parameter for SGD was as follows: *momentum* = 0.0. The default values were used unless otherwise specified.

To evaluate the significance of interchannel information, we trained the model with seven different combinations of channels (i.e., GAD67 + NeuN + Nissl, NeuN + Nissl, GAD67 + Nissl, GAD67 + NeuN, GAD67, NeuN, and Nissl). For each combination, the training lasted 500 epochs, and the model checkpoint method was implemented to prevent the overfitting of the model. When checkpoint methods were applied, the model in the training epoch with the lowest validation loss was saved. Note that the loss is a metric to assess how well the model predicts new data. A batch size of 8 was used for network training. Since our input images were all different in size, the images in a batch were zero-padded to match the size of the largest of the eight images. The model was trained on a GeforceRTX2080Ti GPU (Nvidia, CA, USA), and approximately 1 h was required to complete the training.

Twenty percent of the entire image dataset for either S1 or M1 neurons was set aside as a validation dataset to evaluate the trained models, and the remaining 80% were used as a training-test dataset to train and test the FCN models. To assess the model performance multiple times, 5-fold cross-validations were performed. The training-test data were divided into five segments, and during each training session, one segment was used as test data, while the rest were used as training data.

We calculated the metrics (i.e., accuracy, loss, validation accuracy, and validation loss) and monitored them for each learning epoch. If the validation loss in an epoch was smaller than that in the previous epoch, the weight of the model at that point was saved as a checkpoint. Once the training was finished, the weight of the checkpoint with the lowest validation loss was loaded, and the model was evaluated using the validation dataset.

### Conventional Machine Learning-Based Algorithm

To assess the performance of our deep learning-based algorithm, we additionally classified the cell images using conventional machine learning methods, including principal component analysis (PCA) and the support vector machine (SVM), the combination of which is hereafter called the PCA-SVM model.

After preprocessing, PCA and SVM were implemented using scikit-learn, a Python machine learning library. We used an SVM classifier with a radial basis function kernel, with regularization parameter 1 and balanced class weight. The kernel coefficient (gamma) was calculated as follows.

$$gamma=\frac{1}{\#\;\mathrm{of}\;\mathrm{dimentions}\times \mathrm{sample}\;\mathrm{variance}}.$$

Similar to the deep learning methods, the PCA-SVM model was trained with seven different combinations of channels (i.e., GAD67 + NeuN + Nissl, NeuN + Nissl, GAD67 + Nissl, GAD67 + NeuN, GAD67, NeuN, and Nissl). The trained model was evaluated by 5-fold cross-validations using the same training-test and validation datasets. We first flattened the images into arrays and zero-padded their size to equalize the array size of the largest image. To reduce the dimensionality of the dataset, we applied PCA to the test dataset. Combinations of the components explaining more than 95% of the variance were used as output dimensions. We also put the validation dataset through dimensionality reduction steps by applying the same PCA fit to the training dataset. We trained the SVM classifier on the reduced dataset and evaluated it with the validation data.

### Evaluation of the Model Performance

The performance of the model was evaluated using a weighted F1 score, which takes into account metrics of both GAD67-positive and GAD67-negative neurons. The metrics were defined as follows.

$$precision=\frac{TP}{TP+FN},$$

$$recall=\frac{TP}{TP+FN},$$

$$F1\;score=\frac{2\times precision\times recall}{precision+recall},$$

where TP, FP, TN, and FN represent the true positive, false positive, true negative, and false negative ratios of the classification, respectively. Precision is the ratio of correct positive classification to the total predicted positive classification. The recall is the ratio of correct positive classification to the total true positive samples. The F1 score is a weighted average of precision and recall and can be used as a hybrid metric for evaluation. Furthermore, we calculated a weighted average of the F1 scores in the two classes (i.e., GAD67-positive and GAD67-negative neurons) as follows.

$$weighted\;F1\;score=\frac{N\_cells_{GAD67(+)}\times F1\;score_{GAD67(+)}+N\_cells_{GAD67(-)}\times F1\;score_{GAD67(-)}}{N\_cells_{GAD67(+)}+N\_cells_{GAD67(-)}},$$

where N_cells_GAD67(+)_ and N_cells_GAD67(-)_ represent the number of GAD67-positive and GAD67-negative neurons, respectively.

## Results

### Individual Cell Extraction From Brain Sections

We prepared 100-μm-thick coronal sections from young adult mice. We picked up the slices every 500 μm and immunostained them against NeuN and GAD67. The slices were counterstained with NeuroTrace Nissl stain (hereafter, Nissl) (Figure 1, Supplementary Figure 1). We detected abundant NeuN-immunoreactive (i.e., putatively excitatory) neurons in S1 (Figure 1A) and M1 (Figure 1C) for all three mice tested using a 20× objective lens, thus confirming the presence of GAD67-immunoreactive fluorescence in both cortices (Figures 1B,D). We further confirmed that GAD67-immunoreactive neurons almost overlapped with GABA-immunoreactive neurons (Supplementary Figure 1).

We applied the captured images of NeuN-immunopositive neurons to U-Net (Falk et al., 2019) to detect the contours of single cells (Figure 2). Cell images extracted from 80 and 64 slices from S1 and M1, respectively, were manually labeled GAD67-immunopositive or GAD67-immunonegative by multiple skilled experimenters (Figure 2). For each of the three channels (i.e., GAD67, NeuN, and Nissl), we then calculated the distribution of the average fluorescence intensity of manually annotated GAD67-positive and GAD67-negative neurons in S1 (Figure 3). As expected, GAD67-positive and GAD67-negative neurons exhibited distinct distribution patterns of GAD67 fluorescence (Figure 3A). When the average intensity of GAD67 fluorescence was simply used as a feature for discrimination, the average intensity alone could not necessarily distinguish between the two types of neurons (Figure 3A). However, when using raw images for discrimination, skilled experimenters would have practically integrated more information (underlying the images) and extracted higher-dimensional features to determine the difference between GAD67-positive and GAD67-negative neurons. Neither Nissl nor NeuN fluorescence allowed for discrimination between the two (Figures 3B,C). Moreover, GAD67-positive and GAD67-negative neurons in S1 displayed almost the same unimodal distribution patterns for the sectional area and the circularity of cells (Figures 3D,E). The GAD67-positive and GAD67-negative neurons in M1 also exhibited the same distribution patterns as those in S1 (Figures 3F–J). These results suggest that human-friendly (i.e., easily calculatable and understandable by humans) parameters alone were insufficient for discriminating between GAD67-positive and GAD67-negative cells.

### Deep Learning-Based Automatic Cell Classification

**Figure 5:**
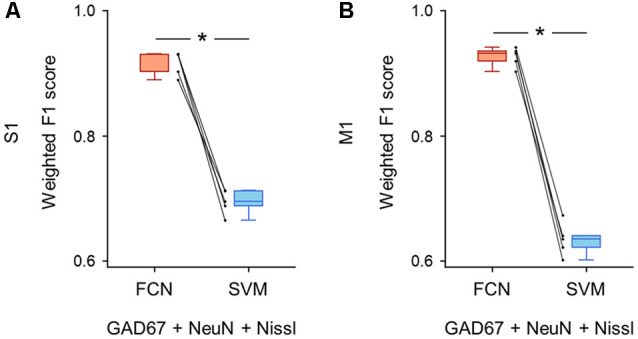
Classification performance of cell images using triple fluorescent signals. **(A)** Weighted F1 score of the FCN model (red) and the PCA-SVM classifier (blue) trained on cell images from S1. All three fluorescent signals (i.e., anti-GAD67, anti-NeuN, and Nissl) were used for training. Each point (gray) signifies the score for the cross-validation (5-fold). *P* = 1.3 × 10^–4^, *t*_(4)_ = 14.7, *n* = 5-fold cross-validations, paired *t*-test. *P < 0.05. **(B)** The same as **(A)**, but for M1. *P* = 1.4 × 10^–5^, *t*_(4)_ = 25.6, *n* = 5-fold cross-validations, paired *t*-test. *P < 0.05. Abbreviations: S1, primary somatosensory cortex; M1, primary motor cortex; FCN, fully convolutional network; SVM, support vector machine; PCA, principal component analysis.

**Figure 6:**
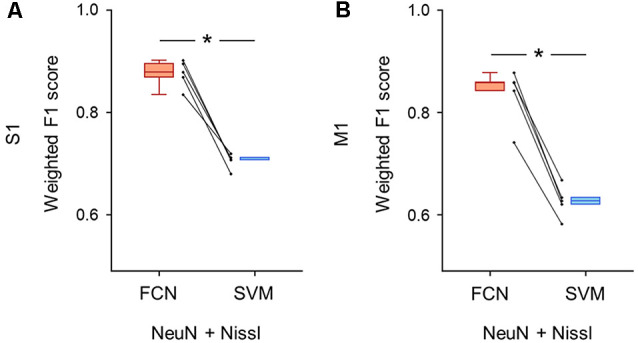
Classification performance of cell images using both anti-NeuN and Nissl fluorescence signals. **(A)** Weighted F1 score of the FCN model (red) and the PCA-SVM classifier (blue) trained on cell images from S1. Two fluorescent signals (i.e., anti-NeuN and Nissl) were used for training. Each point (gray) signifies the score for the cross-validation (5-fold). *P* = 3.0 × 10^–4^, *t*_(4)_ = 11.7, *n* = 5-fold cross-validations, paired *t*-test. *P < 0.05. **(B)** The same as **(A)**, but for M1. *P* = 1.9 × 10^–4^, *t*_(4)_ = 13.2, *n* = 5-fold cross-validations, paired *t*-test. *P < 0.05. Abbreviations: S1, primary somatosensory cortex; M1, primary motor cortex; FCN, fully convolutional network; SVM, support vector machine; PCA, principal component analysis.

**Figure 7:**
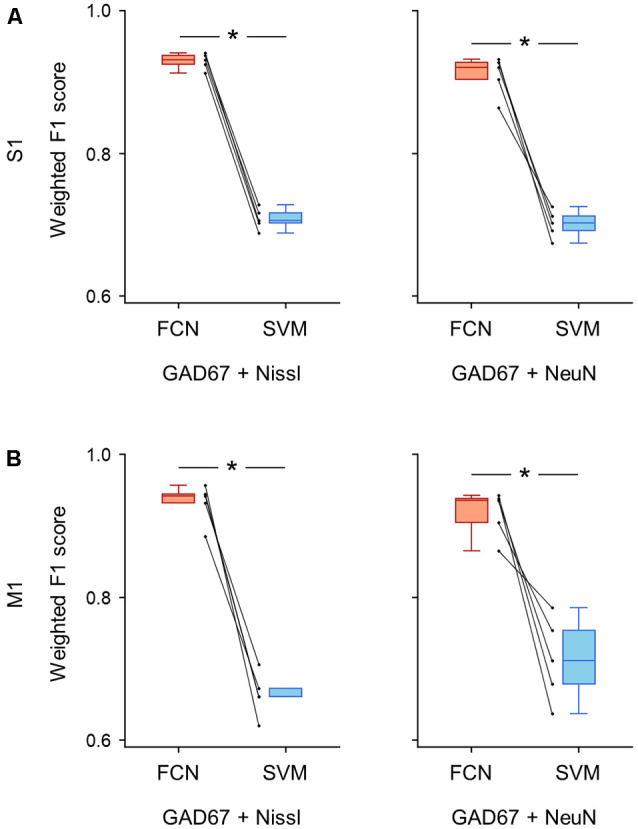
Classification performance of cell images using signal pairs including anti-GAD67 fluorescence. **(A)** Weighted F1 score of the FCN model (red) and the PCA-SVM classifier (blue) trained on cell images from S1. Each point (gray) signifies the score for cross-validation (5-fold). Left: classification performance using anti-GAD67 and Nissl fluorescence signals. *P* = 9.9 × 10^–7^, *t*_(4)_ = 46.9, *n* = 5-fold cross-validations, paired *t*-test. *P < 0.05. Right: the same as on the left, but for anti-GAD67 and anti-NeuN immunofluorescence signals. *P* = 4.1 × 10^–4^, *t*_(4)_ = 10.8, *n* = 5-fold cross-validations, paired *t*-test. *P < 0.05. **(B)** The same as **(A)**, but for M1. Left: *P* = 2.8 × 10^–4^, *t*_(4)_ = 12.0, *n* = 5-fold cross-validations, paired *t*-test. *P < 0.05. Right: *P* = 6.8 × 10^–3^, *t*_(4)_ = 5.1, *n* = 5-fold cross-validations, paired *t*-test. *P < 0.05. Abbreviations: S1, primary somatosensory cortex; M1, primary motor cortex; FCN, fully convolutional network; SVM, support vector machine; PCA, principal component analysis.

**Figure 8:**
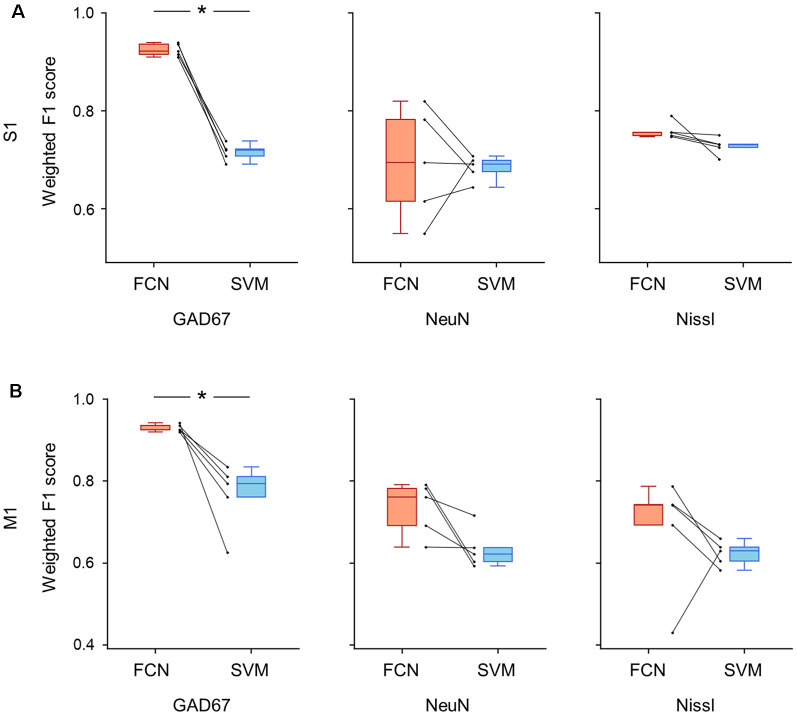
Classification performance of cell images using single fluorescent signals. **(A)** Weighted F1 score of the FCN model (red) and the PCA-SVM classifier (blue) trained on cell images from S1. Left: classification performance using an anti-GAD67 signal. Each point (gray) signifies the score for the cross-validation (5-fold). *P* = 5.6 × 10^–5^, *t*_(4)_ = 18.0, *n* = 5-fold cross-validations, paired *t*-test. *P < 0.05. Middle, the same as on the left, but for an anti-NeuN signal. *P* = 0.87, *t*_(4)_ = 0.18, *n* = 5-fold cross-validations, paired *t*-test. Right: the same as on the left, but for a Nissl signal. *P* = 0.09, *t*_(4)_ = 2.18, *n* = 5-fold cross-validations, paired *t*-test. **(B)** The same as **(A)**, but for M1. Left, *P* = 0.01, *t*_(4)_ = 4.1, *n* = 5-fold cross-validations, paired *t*-test. *P < 0.05. Middle, *P* = 0.06, *t*_(4)_ = 2.6, *n* = 5-fold cross-validations, paired *t*-test. Right, *P* = 0.22, *t*_(4)_ = 1.45, *n* = 5-fold cross-validations, paired *t*-test. Abbreviations: S1, primary somatosensory cortex; M1, primary motor cortex; FCN, fully convolutional network; SVM, support vector machine; PCA, principal component analysis.

Simple quantitative metrics (i.e., fluorescence intensity, sectional area, and circularity) did not match the manual annotations of GAD67-positive and GAD67-negative cells as defined by skilled experimenters; therefore, we implemented FCN to automatically classify individual cell images into GAD67-positive and GAD67-negative neurons (Figure 4). To evaluate the performance of the deep learning-based method, we introduced a classification metric called the weighted F1 score and compared it between the FCN and the PCA-SVM classifiers (Figures 5–8). The weighted F1 score was calculated for each combination of channels (e.g., GAD67 + NeuN + Nissl (Figure 5), NeuN + Nissl (Figure 6), GAD67 + Nissl, GAD67 + NeuN (Figure 7), GAD67, NeuN and Nissl (Figure 8)) for S1 and M1.

First, we applied the images with all three channels (i.e., GAD67 + NeuN + Nissl) to the FCN-based model and the PCA-SVM classifier and evaluated their classification performance. The FCN model classified the cortical cells into GAD67-positive and GAD67-negative cortical cells with high F1 scores in both brain regions (S1: 0.92 ± 0.02; M1: 0.92 ± 0.01; Supplementary Table). The accuracy of the PCA-SVM classifier was above the chance level (S1: 0.70 ± 0.02; M1: 0.63 ± 0.02; Supplementary Table). The weighted F1 score of the FCN model was significantly higher than that of the PCA-SVM classifier in S1 (*P* = 1.26 × 10^–4^, *t*_(4)_ = 14.7, *n* = 5-fold cross-validations, paired *t*-test; Figure 5A) and M1 (*P* = 1.38 × 10^–5^, *t*_(4)_ = 25.6, *n* = 5-fold cross-validations, paired *t*-test; Figure 5B).

We trained the FCN model and the PCA-SVM classifier on images without the GAD67 channel to test whether the classification of GAD67-positive and GAD67-negative neurons was achieved by Nissl and NeuN fluorescence. The FCN model using the two channels still performed well with slightly decreased accuracy compared to using all the channels (S1: 0.88 ± 0.02; M1: 0.84 ± 0.05; Supplementary Table). Moreover, the weighted F1 score with the FCN model was significantly higher than that with the PCA-SVM classifier (S1: 0.70 ± 0.01; M1: 0.63 ± 0.03; Supplementary Table) in both S1 (*P* = 3.04 × 10^–4^, *t*_(4)_ = 11.7, *n* = 5-fold cross-validations, paired *t*-test; Figure 6A) and M1 (*P* = 1.91 × 10^–4^, *t*_(4)_ = 13.2, *n* = 5-fold cross-validations, paired *t*-test; Figure 6B).

To further visualize the performance of the model trained without the GAD67 immunosignals, we displayed the distribution of average fluorescence intensities of the validation dataset for each of the three channels (i.e., GAD67, NeuN, and Nissl); note that the validation dataset included manually annotated and deep learning-predicted GAD67-positive/negative neurons in S1 and M1 (Supplementary Figures 2, 3). There were no apparent differences in the distributions of any parameters (i.e., GAD67 fluorescence, NeuN fluorescence, Nissl fluorescence, ROI area, and ROI circularity) between manually annotated and deep learning-predicted GAD67-positive/negative neurons in either brain region.

We calculated the evaluation metrics for the channel combinations, including the GAD67 channel (i.e., GAD67 + Nissl, GAD67 + NeuN). As expected, the FCN models trained on these combinations performed well on the basis of the weighted F1 score compared to the PCA-SVM classifier for S1 (GAD67 + Nissl: 0.93 ± 0.01 (FCN) *vs*. 0.71 ± 0.01 (PCA-SVM), *P* = 9.87 × 10^–7^, *t*_(4)_ = 46.9, *n* = 5-fold cross-validations, paired *t*-test; GAD67 + NeuN: 0.91 ± 0.02 (FCN) *vs*. 0.70 ± 0.02 (PCA-SVM), *P* = 4.12 × 10^–4^, *t*_(4)_ = 10.8, *n* = 5-fold cross-validations, paired *t*-test; Figure 7A; Supplementary Table) and M1 (GAD67 + Nissl: 0.93 ± 0.03 (FCN) *vs*. 0.66 ± 0.03 (PCA-SVM), *P* = 2.78 × 10^–4^, *t*_(4)_ = 12.0, *n* = 5-fold cross-validations, paired *t*-test; GAD67 + NeuN: 0.92 ± 0.03 (FCN) *vs*. 0.71 ± 0.05 (PCA-SVM), *P* = 6.83 × 10^–3^, *t*_(4)_ = 5.13, *n* = 5-fold cross-validations, paired *t*-test; Figure 7B, Supplementary Table).

We further trained the FCN model and the SVM classifier on single channels (i.e., Nissl only, NeuN only, and GAD67 only) and evaluated their performances using the weighted F1 scores for S1 and M1 (Figure 8). When the models were trained using the GAD67 channel alone, the performance of the FCN model was high (>0.9) for both cortical regions compared to the conventional PCA-SVM method (S1: 0.93 ± 0.01 (FCN) vs. 0.72 ± 0.02 (PCA-SVM), *P* = 5.62 × 10^–5^, *t*_(4)_ = 18.0, *n* = 5-fold cross-validations, paired *t*-test; Figure 8A, Supplementary Table; M1: 0.93 ± 0.01 (FCN) vs. 0.77 ± 0.07 (PCA-SVM), *P* = 1.44 × 10^–2^, *t*_(4)_ = 4.13, *n* = 5-fold cross-validations, paired *t*-test; Figure 8B, Supplementary Table). The weighted F1 scores of the FCN models trained on other single channels (i.e., NeuN only or Nissl only) did not significantly differ from those of the PCA-SVM classifiers for S1 (Nissl: 0.69 ± 0.1 (FCN) vs. 0.68 ± 0.02 (PCA-SVM), *P* = 0.09, *t*_(4)_ = 2.19, *n* = 5-fold cross-validations, paired *t*-test; NeuN: 0.76 ± 0.02 (FCN) vs. 0.71 ± 0.02 (PCA-SVM), *P* = 0.86, *t*_(4)_ = 0.18, *n* = 5-fold cross-validations, paired *t*-test; Figure 8A, Supplementary Table) or M1 (Nissl: 0.73 ± 0.06 (FCN) *vs*. 0.63 ± 0.04 (PCA-SVM), *P* = 0.44, *t*_(4)_ = 0.85, *n* = 5-fold cross-validations, paired *t*-test; NeuN: 0.70 ± 0.13 (FCN) *vs*. 0.62 ± 0.03 (PCA-SVM), *P* = 0.06, *t*_(4)_ = 2.58, *n* = 5-fold cross-validations, paired *t*-test; Figure 8B, Supplementary Table), suggesting that the single channel information of either NeuN or Nissl is insufficient for automatic discrimination between GAD67-positive and GAD67-negative neurons.

## Discussion

The deep neural network-based algorithm established in this study classified GAD67-positive and GAD67-negative cells with high accuracy. In the presence of the GAD67 fluorescence channel, the weighted F1 score of our FCN model was significantly higher than that of the PCA-SVM hybrid model. Surprisingly, even in the absence of the GAD67 channel, the FCN model exhibited higher performance than the PCA-SVM classifier.

Intuitively, the high classification performance of the FCN model using all three channels was not surprising (Figures 5–8) because the cortical neurons were labeled by human experimenters based primarily on GAD67 fluorescence. Nevertheless, this result suggests that our FCN model can predict the neuron subtypes with near-human accuracy when the network is fed with the GAD67 channel.

Moreover, even without the GAD67 channel, the performance of the FCN model was superior to that of the PCA-SVM classifier (Figure 6). Although the classification metrics of the FCN model trained on both the Nissl and NeuN channels were not as high as those of the model trained on all three channels (i.e., GAD67 + NeuN + Nissl), the metrics for the two (i.e., Nissl and NeuN) channels were high enough for practical use. The requirement for both NeuN and Nissl fluorescence also indicates that there are morphological and neurochemical features in the NeuN- and/or Nissl-stained images that can be distinguished by the fully trained FCN model. Strictly, the features extracted from the cell images by the neural network are obscure for humans, although one possible anatomical characteristic learned by the neural network may be cell type-dependent expression patterns of NeuN. Abundant NeuN expression within cell nuclei is associated with the increased expression of transcripts encoding marker proteins for neurogenesis and neuroplasticity and chromatin-modifying enzymes regulating histone acetylation and methylation (Yu et al., 2015). During brain development, one of the chromatin-modifying enzymes, HDAC1 (histone deacetylase 1), binds to parvalbumin promoters and thereby downregulates the gene expression required for the maturation of parvalbumin-positive interneurons (Koh and Sng, 2016). These results are potentially reflected by our findings that GAD67-positive cells exhibited relatively low NeuN expression (Figure 3).

Interestingly, however, the classification performance of the FCN pre-trained on either the NeuN or Nissl fluorescence channel was not significantly different from that of the conventional method, indicating that both fluorescence channels are necessary for the FCN model to perform better than the PCA-SVM model in terms of classification of GAD67-positive and GAD67-negative cells. The difference in the classification score of the FCN model between single-channel training (i.e., either Nissl or NeuN) and channel-pair training (i.e., both Nissl and NeuN) may stem from the close relationship between the fluorescence intensity of the two channels. These results are consistent with our hypothesis that cortical neurons are classified based on anti-NeuN and Nissl signals. This relationship is undetectable by humans but mined by the deep neural network. Information on the single fluorescence channel is insufficient for the classification of GAD67-positive and GAD67-negative cells regardless of whether the fluorescence intensity is based on the characteristics of cells or dependent on the focus depth during image acquisition.

During this study, the FCN models performed even better than the PCA-SVM models. Compared with the SVM, the advantage of the FCN lies in its ability to optimize better with relatively small datasets (e.g., images), especially when the number of parameters (e.g., pixels) is large (Hasan et al., 2019). However, when the SVM is used for binary classification, it generally needs a greater number of data than parameters. Given that high-resolution images (composed of many pixels) were analyzed, we used PCA to reduce the parameter size and applied SVM to the data with reduced dimensions. In consideration of the significant effect of the data size on the performance of the SVM, the number of datasets in the current study may have been insufficient for high performance.

We consider that our deep neural network had several advantages compared with previous implementations of deep learning. (i) Although training a deep network for image segmentation is a popular method for the detection and classification of tissue sections in images (Sadanandan et al., 2017; Al-Kofahi et al., 2018), previous methods are generally composed of an encoder and a decoder. These deep networks usually require more convolutional layers than a simple FCN model (Ronneberger et al., 2015) and are accompanied by more learning parameters, allowing for less complicated modifications (Xiao et al., 2018). (ii) Our workflow uses a pre-trained network to extract cell images from the segmented image and learns their features with only three convolutional layers. Thus, our simple method saves time for the optimization of multiple hyperparameters and thus can be run by a normal laboratory computer. (iii) The advantage of the FCN model lies in the lack of a fully connected layer. With the fully connected layer alone, the input array is expected to be the same size; thus, without this layer, the network can accept images of virtually any size. (iv) By downsampling the image dataset of GAD67-positive and GAD67-negative neurons, we would expect a faster convergence of the model and save computer disk memory. Therefore, the current method outperforms the previous deep learning-based method for cell classification.

Our novel deep learning-based method of detecting GAD67-expressing neurons has the potential to provide the unbiased classification of interneurons in the neocortex, where neurons are distributed ubiquitously and sparsely. In contrast to the neocortex, the hippocampus is composed of the pyramidal cell layer (stratum pyramidale) and the other layers (including stratum oriens, stratum radiatum, and stratum lacunosum moleculare). Excitatory neurons are densely packed in the pyramidal cell layer, whereas inhibitory neurons are sparsely distributed in the other layers (Supplementary Figure 4). Our FCN model may not be suitable for the hippocampal pyramidal cell layer because a densely packed neuronal population precludes us from precisely separating cells one-by-one (Figure 2). On the other hand, most neurons in the other layers in the hippocampus were inhibitory and distributed more sparsely than any layer in the neocortex (Supplementary Figure 4). Thus, inhibitory neurons would be easily detected even without our deep learning-based model.

As shown in various studies, interneurons have a vast diversity of structural and functional features (Lawrence and McBain, 2003; Maccaferri and Lacaille, 2003; Whittington and Traub, 2003; Buzsáki et al., 2004; Monyer and Markram, 2004; Melzer and Monyer, 2020). This enormous diversity of cortical interneurons plays a significant role in increasing and expanding the computational power of neural circuits but at the same time, poses a challenge for understanding their functions (Tremblay et al., 2016). Cortical interneurons are typically classified based on their morphological, neurochemical, and physiological characteristics; however, the interneurons differ over more parameters (The Petilla Interneuron Nomenclature Group, 2008). Deep learning may be beneficial and practical for the unbiased classification of the interneuron subtypes from the perspectives of morphology and neurochemistry.

A previous study demonstrated that convolutional neural networks could morphologically profile cell types (Pawlowski et al., 2016). Generally, the mechanism underlying deep neural network-based classification is regarded as a black box. It is almost difficult for humans to understand how deep learning classifies cell types by eye (Supplementary Figure 5). However, there are several methods to visualize what the deep neural network focuses on when performing classifications (Erhan et al., 2009; Simonyan et al., 2013). When applied to our trained FCN model, this visualization may reveal features that humans have overlooked. In this sense, the current model will open the door to uncovering unbiased and novel criteria for neuron-type classification.

## Data Availability Statement

The datasets presented in this study can be found in online repositories. The names of the repository/repositories and accession number(s) can be found below: https://bit.ly/2OkGscV.

## Ethics Statement

The animal study was reviewed and approved by the Fundamental Guidelines for the Proper Conduct of Animal Experiments and Related Activities in Academic Research Institutions (Ministry of Education, Culture, Sports, Science and Technology, Notice No. 71 of 2006), the Standards for Breeding and Housing of and Pain Alleviation for Experimental Animals (Ministry of the Environment, Notice No. 88 of 2006) and the Guidelines on the Method of Animal Disposal (Prime Minister’s Office, Notice No. 40 of 1995).

## Author Contributions

KY, NM, and YI conceptualized the research. KY and NM performed the experiments. KY, JL, and NM analyzed the data. KY, JL, NM, and YI discussed the project and wrote the manuscript by mutual consent. All authors contributed to the article and approved the submitted version.

## Conflict of Interest

The authors declare that the research was conducted in the absence of any commercial or financial relationships that could be construed as a potential conflict of interest.

## Abbreviations

NeuN: neuronal nuclei
GAD67: glutamate decarboxylase 67
GABA: gamma-aminobutyric acid
S1: primary somatosensory cortex
M1: primary motor cortex
ROI: region of interest
FCN: fully convolutional network
TP: true positive
FP: false positive
TN: true negative
FN: false negative
PCA: principal component analysis
SVM: support vector machine.
