# Supplementary material for: Deep Learning-Based Classification of GAD67-Positive Neurons Without the Immunosignal
Source: Front Neuroanat. 2021 Mar 31;15:643067. doi: 10.3389/fnana.2021.643067 (PMC8044854; doi:10.3389/fnana.2021.643067)
Supplement: Supplementary file 1 [file Data_Sheet_1.pdf]

Yamashiro *et al.*, Supplementary Materials

Contents

- Supplementary Table 1
 Metrics of classification performance of the FCN-based model for S1.
Pages 1-2

- Supplementary Table 2
 Metrics of classification performance of the PCA-SVM model for S1.
Pages 3-4

- Supplementary Table 3
 Metrics of classification performance of the FCN-based model for M1.
Pages 5-6

- Supplementary Table 4
 Metrics of classification performance of the PCA-SVM model for M1.
Pages 7-8

- Supplementary Figure 1
 Representative photographs of GAD67 and GABA immunoreactivity in S1 and M1.
Page 9

- Supplementary Figure 2
 Differences in the immunofluorescence and shape of GAD67-positive and GAD67-negative cells in S1 as manually annotated or predicted by deep learning.
Page 10

- Supplementary Figure 3
 Differences in the immunofluorescence and shape of GAD67-positive and GAD67-negative cells in M1 as manually annotated or predicted by deep learning.
Page 11

- Supplementary Figure 4
 Representative photographs of GAD67 and NeuN immunoreactivity and Nissl reactivity in the hippocampal CA1 area.
Page 12

- Supplementary Figure 5
 Representative photographs of GAD67 and NeuN immunoreactivity and Nissl reactivity in single neocortical cells categorized by experimenters and deep learning.
Pages 13-14

Abbreviations: S1, primary somatosensory cortex; M1, primary motor cortex; FCN, fully convolutional network; PCA, principal component analysis; SVM, support vector machine.

A S1 FCN GAD67 + NeuN + Nissl

Cross validation	Accuracy	GAD67(-) precision	GAD67(-) recall	GAD67(-) F1 score	GAD67(+) precision	GAD67(+) recall	GAD67(+) F1 score	Macro precision	Macro recall	Macro F1 score	Weighted precision	Weighted recall	Weighted F1 score
1	0.910	0.998	0.908	0.951	0.321	0.950	0.480	0.659	0.929	0.715	0.968	0.910	0.930
2	0.868	0.998	0.863	0.926	0.245	0.970	0.391	0.622	0.917	0.659	0.965	0.868	0.903
3	0.910	0.998	0.908	0.950	0.320	0.950	0.479	0.659	0.929	0.715	0.968	0.910	0.930
4	0.848	0.999	0.842	0.914	0.221	0.981	0.361	0.610	0.911	0.637	0.965	0.848	0.890
5	0.912	0.997	0.911	0.952	0.324	0.940	0.482	0.661	0.925	0.717	0.968	0.912	0.931
Average	0.890	0.998	0.886	0.939	0.286	0.958	0.439	0.642	0.922	0.689	0.967	0.890	0.917

B S1 FCN NeuN + Nissl

Cross validation	Accuracy	GAD67(-) precision	GAD67(-) recall	GAD67(-) F1 score	GAD67(+) precision	GAD67(+) recall	GAD67(+) F1 score	Macro precision	Macro recall	Macro F1 score	Weighted precision	Weighted recall	Weighted F1 score
1	0.818	0.991	0.817	0.896	0.173	0.840	0.287	0.582	0.828	0.591	0.955	0.818	0.869
2	0.859	0.990	0.861	0.921	0.209	0.803	0.332	0.600	0.832	0.627	0.956	0.859	0.895
3	0.834	0.991	0.835	0.906	0.186	0.829	0.304	0.589	0.832	0.605	0.956	0.834	0.880
4	0.767	0.992	0.763	0.863	0.143	0.868	0.246	0.568	0.816	0.554	0.955	0.767	0.836
5	0.870	0.989	0.874	0.928	0.222	0.784	0.346	0.605	0.829	0.637	0.955	0.870	0.903
Average	0.830	0.991	0.830	0.903	0.187	0.825	0.303	0.589	0.828	0.603	0.955	0.830	0.876

C S1 FCN GAD67 + Nissl

Cross validation	Accuracy	GAD67(-) precision	GAD67(-) recall	GAD67(-) F1 score	GAD67(+) precision	GAD67(+) recall	GAD67(+) F1 score	Macro precision	Macro recall	Macro F1 score	Weighted precision	Weighted recall	Weighted F1 score
1	0.883	0.997	0.880	0.935	0.265	0.944	0.414	0.631	0.912	0.674	0.965	0.883	0.912
2	0.927	0.994	0.930	0.961	0.361	0.868	0.510	0.677	0.899	0.735	0.966	0.927	0.941
3	0.921	0.995	0.922	0.957	0.344	0.890	0.496	0.669	0.906	0.727	0.966	0.921	0.937
4	0.912	0.996	0.912	0.952	0.322	0.911	0.476	0.659	0.912	0.714	0.966	0.912	0.931
5	0.903	0.997	0.901	0.947	0.303	0.935	0.457	0.650	0.918	0.702	0.966	0.903	0.925
Average	0.909	0.996	0.909	0.950	0.319	0.910	0.471	0.657	0.909	0.710	0.966	0.909	0.929

D S1 FCN GAD67 + NeuN

Cross validation	Accuracy	GAD67(-) precision	GAD67(-) recall	GAD67(-) F1 score	GAD67(+) precision	GAD67(+) recall	GAD67(+) F1 score	Macro precision	Macro recall	Macro F1 score	Weighted precision	Weighted recall	Weighted F1 score
1	0.913	0.996	0.913	0.953	0.326	0.927	0.483	0.661	0.920	0.718	0.967	0.913	0.932
2	0.906	0.996	0.905	0.949	0.309	0.924	0.463	0.652	0.915	0.706	0.966	0.906	0.927
3	0.810	0.998	0.802	0.890	0.184	0.972	0.309	0.591	0.887	0.599	0.963	0.810	0.864
4	0.896	0.996	0.894	0.943	0.286	0.927	0.437	0.641	0.910	0.690	0.965	0.896	0.920
5	0.870	0.997	0.867	0.927	0.245	0.944	0.389	0.621	0.905	0.658	0.964	0.870	0.904
Average	0.879	0.997	0.876	0.932	0.270	0.939	0.416	0.633	0.907	0.674	0.965	0.879	0.910

Supplementary Table 1. Metrics of classification performance of the FCN-based model for S1 **(continued on the next page).**

E S1 FCN GAD67

Cross validation	Accuracy	GAD67(-) precision	GAD67(-) recall	GAD67(-) F1 score	GAD67(+) precision	GAD67(+) recall	GAD67(+) F1 score	Macro precision	Macro recall	Macro F1 score	Weighted precision	Weighted recall	Weighted F1 score
1	0.882	0.990	0.885	0.935	0.241	0.795	0.370	0.615	0.840	0.652	0.957	0.882	0.910
2	0.900	0.991	0.904	0.945	0.280	0.816	0.417	0.636	0.860	0.681	0.960	0.900	0.922
3	0.889	0.993	0.890	0.939	0.265	0.870	0.406	0.629	0.880	0.672	0.962	0.889	0.915
4	0.921	0.992	0.925	0.957	0.337	0.834	0.480	0.664	0.879	0.719	0.963	0.921	0.936
5	0.925	0.991	0.930	0.960	0.349	0.816	0.489	0.670	0.873	0.724	0.963	0.925	0.939
Average	0.903	0.991	0.907	0.947	0.294	0.826	0.432	0.643	0.867	0.690	0.961	0.903	0.925

F S1 FCN NeuN

Cross validation	Accuracy	GAD67(-) precision	GAD67(-) recall	GAD67(-) F1 score	GAD67(+) precision	GAD67(+) recall	GAD67(+) F1 score	Macro precision	Macro recall	Macro F1 score	Weighted precision	Weighted recall	Weighted F1 score
1	0.490	0.995	0.469	0.638	0.076	0.948	0.140	0.535	0.709	0.389	0.955	0.490	0.616
2	0.692	0.990	0.685	0.810	0.109	0.844	0.193	0.549	0.765	0.502	0.951	0.692	0.783
3	0.422	0.994	0.399	0.569	0.067	0.946	0.125	0.530	0.672	0.347	0.953	0.422	0.550
4	0.744	0.989	0.740	0.847	0.127	0.823	0.219	0.558	0.782	0.533	0.951	0.744	0.819
5	0.579	0.993	0.563	0.719	0.087	0.914	0.159	0.540	0.738	0.439	0.953	0.579	0.694
Average	0.585	0.992	0.571	0.716	0.093	0.895	0.167	0.543	0.733	0.442	0.953	0.585	0.692

G S1 FCN Nissl

Cross validation	Accuracy	GAD67(-) precision	GAD67(-) recall	GAD67(-) F1 score	GAD67(+) precision	GAD67(+) recall	GAD67(+) F1 score	Macro precision	Macro recall	Macro F1 score	Weighted precision	Weighted recall	Weighted F1 score
1	0.702	0.978	0.704	0.819	0.092	0.657	0.162	0.535	0.681	0.490	0.939	0.702	0.790
2	0.655	0.979	0.653	0.784	0.084	0.695	0.150	0.532	0.674	0.467	0.940	0.655	0.756
3	0.655	0.981	0.653	0.784	0.086	0.719	0.154	0.534	0.686	0.469	0.942	0.655	0.756
4	0.647	0.981	0.644	0.777	0.085	0.724	0.152	0.533	0.684	0.465	0.942	0.647	0.750
5	0.644	0.980	0.641	0.775	0.083	0.711	0.149	0.531	0.676	0.462	0.941	0.644	0.748
Average	0.661	0.980	0.659	0.788	0.086	0.701	0.153	0.533	0.680	0.471	0.941	0.661	0.760

Supplementary Table 1. Metrics of classification performance of the FCN-based model for S1.
A, Performance of the FCN-based model using anti-GAD67, anti-NeuN, and Nissl signals in S1. ***B***, The same as ***A***, but using anti-NeuN and Nissl signals. ***C***, The same as ***A***, but using anti-GAD67 and Nissl signals. ***D***, The same as ***A***, but using anti-GAD67 and anti-NeuN signals. ***E***, The same as ***A***, but using an anti-GAD67 signal. ***F***, The same as ***A***, but using an anti-NeuN signal. ***G***, The same as ***A***, but using a Nissl signal. *Abbreviations*: S1, primary somatosensory cortex; FCN, fully convolutional network.

A S1 SVM GAD67 + NeuN + Nissl

Cross validation	Accuracy	GAD67(-) precision	GAD67(-) recall	GAD67(-) F1 score	GAD67(+) precision	GAD67(+) recall	GAD67(+) F1 score	Macro precision	Macro recall	Macro F1 score	Weighted precision	Weighted recall	Weighted F1 score
1	0.539	0.961	0.540	0.692	0.049	0.523	0.090	0.505	0.531	0.391	0.921	0.539	0.665
2	0.575	0.962	0.579	0.723	0.052	0.503	0.094	0.507	0.541	0.408	0.922	0.575	0.695
3	0.596	0.961	0.602	0.740	0.051	0.471	0.093	0.506	0.536	0.416	0.922	0.596	0.712
4	0.598	0.960	0.605	0.742	0.050	0.454	0.090	0.505	0.529	0.416	0.921	0.598	0.713
5	0.567	0.960	0.571	0.716	0.049	0.484	0.089	0.505	0.527	0.403	0.920	0.567	0.689
Average	0.575	0.961	0.579	0.723	0.050	0.487	0.091	0.506	0.533	0.407	0.921	0.575	0.695

B S1 SVM NeuN + Nissl

Cross validation	Accuracy	GAD67(-) precision	GAD67(-) recall	GAD67(-) F1 score	GAD67(+) precision	GAD67(+) recall	GAD67(+) F1 score	Macro precision	Macro recall	Macro F1 score	Weighted precision	Weighted recall	Weighted F1 score
1	0.557	0.959	0.561	0.708	0.047	0.475	0.086	0.503	0.518	0.397	0.919	0.557	0.681
2	0.591	0.961	0.596	0.736	0.051	0.475	0.092	0.506	0.536	0.414	0.922	0.591	0.708
3	0.597	0.961	0.603	0.741	0.050	0.460	0.091	0.506	0.532	0.416	0.921	0.597	0.713
4	0.607	0.961	0.614	0.749	0.050	0.449	0.091	0.506	0.531	0.420	0.921	0.607	0.720
5	0.591	0.960	0.597	0.736	0.048	0.449	0.088	0.504	0.523	0.412	0.920	0.591	0.708
Average	0.588	0.960	0.594	0.734	0.050	0.462	0.089	0.505	0.528	0.412	0.920	0.588	0.706

C S1 SVM GAD67 + Nissl

Cross validation	Accuracy	GAD67(-) precision	GAD67(-) recall	GAD67(-) F1 score	GAD67(+) precision	GAD67(+) recall	GAD67(+) F1 score	Macro precision	Macro recall	Macro F1 score	Weighted precision	Weighted recall	Weighted F1 score
1	0.567	0.961	0.570	0.716	0.049	0.488	0.090	0.505	0.529	0.403	0.921	0.567	0.688
2	0.588	0.962	0.593	0.734	0.052	0.490	0.094	0.507	0.542	0.414	0.922	0.588	0.706
3	0.617	0.961	0.625	0.757	0.051	0.443	0.092	0.506	0.534	0.425	0.921	0.617	0.728
4	0.602	0.961	0.608	0.745	0.051	0.458	0.091	0.506	0.533	0.418	0.921	0.602	0.716
5	0.584	0.960	0.589	0.730	0.049	0.467	0.089	0.505	0.528	0.410	0.920	0.584	0.702
Average	0.592	0.961	0.597	0.736	0.051	0.469	0.091	0.506	0.533	0.414	0.921	0.592	0.708

D S1 SVM GAD67 + NeuN

Cross validation	Accuracy	GAD67(-) precision	GAD67(-) recall	GAD67(-) F1 score	GAD67(+) precision	GAD67(+) recall	GAD67(+) F1 score	Macro precision	Macro recall	Macro F1 score	Weighted precision	Weighted recall	Weighted F1 score
1	0.550	0.961	0.552	0.701	0.049	0.508	0.090	0.505	0.530	0.395	0.921	0.550	0.674
2	0.585	0.962	0.589	0.731	0.051	0.486	0.093	0.506	0.538	0.412	0.922	0.585	0.703
3	0.613	0.961	0.621	0.754	0.051	0.447	0.092	0.506	0.534	0.423	0.921	0.613	0.725
4	0.596	0.961	0.602	0.740	0.051	0.471	0.093	0.506	0.536	0.416	0.922	0.596	0.712
5	0.571	0.960	0.575	0.719	0.049	0.482	0.089	0.505	0.528	0.404	0.921	0.571	0.692
Average	0.583	0.961	0.588	0.729	0.050	0.479	0.091	0.506	0.533	0.410	0.921	0.583	0.701

Supplementary Table 2. Metrics of classification performance of the PCA-SVM model for S1 (continued on the next page).

E S1 SVM GAD67

Cross validation	Accuracy	GAD67(-) precision	GAD67(-) recall	GAD67(-) F1 score	GAD67(+) precision	GAD67(+) recall	GAD67(+) F1 score	Macro precision	Macro recall	Macro F1 score	Weighted precision	Weighted recall	Weighted F1 score
1	0.591	0.961	0.597	0.736	0.050	0.467	0.091	0.505	0.532	0.413	0.921	0.591	0.708
2	0.609	0.962	0.616	0.751	0.053	0.469	0.095	0.507	0.542	0.423	0.922	0.609	0.722
3	0.631	0.962	0.639	0.768	0.053	0.441	0.094	0.507	0.540	0.431	0.922	0.631	0.739
4	0.606	0.961	0.613	0.748	0.051	0.458	0.092	0.506	0.535	0.420	0.921	0.606	0.720
5	0.571	0.959	0.576	0.720	0.047	0.460	0.086	0.503	0.518	0.403	0.919	0.571	0.692
Average	0.602	0.961	0.608	0.745	0.051	0.459	0.092	0.506	0.533	0.418	0.921	0.602	0.716

F S1 SVM NeuN

Cross validation	Accuracy	GAD67(-) precision	GAD67(-) recall	GAD67(-) F1 score	GAD67(+) precision	GAD67(+) recall	GAD67(+) F1 score	Macro precision	Macro recall	Macro F1 score	Weighted precision	Weighted recall	Weighted F1 score
1	0.515	0.960	0.514	0.670	0.048	0.531	0.087	0.504	0.523	0.379	0.920	0.515	0.644
2	0.552	0.962	0.553	0.703	0.051	0.527	0.093	0.507	0.540	0.398	0.923	0.552	0.676
3	0.580	0.960	0.585	0.727	0.049	0.469	0.089	0.505	0.527	0.408	0.920	0.580	0.699
4	0.591	0.961	0.596	0.736	0.050	0.469	0.091	0.506	0.533	0.414	0.921	0.591	0.708
5	0.570	0.959	0.575	0.719	0.048	0.467	0.087	0.504	0.521	0.403	0.919	0.570	0.691
Average	0.562	0.961	0.565	0.711	0.049	0.492	0.089	0.505	0.529	0.400	0.921	0.562	0.684

G S1 SVM Nissl

Cross validation	Accuracy	GAD67(-) precision	GAD67(-) recall	GAD67(-) F1 score	GAD67(+) precision	GAD67(+) recall	GAD67(+) F1 score	Macro precision	Macro recall	Macro F1 score	Weighted precision	Weighted recall	Weighted F1 score
1	0.583	0.959	0.589	0.730	0.047	0.447	0.086	0.503	0.518	0.408	0.919	0.583	0.701
2	0.621	0.960	0.631	0.761	0.050	0.421	0.089	0.505	0.526	0.425	0.920	0.621	0.732
3	0.646	0.960	0.658	0.781	0.051	0.402	0.090	0.505	0.530	0.435	0.920	0.646	0.750
4	0.621	0.959	0.631	0.761	0.048	0.404	0.085	0.503	0.517	0.423	0.919	0.621	0.731
5	0.614	0.959	0.623	0.755	0.048	0.413	0.085	0.503	0.518	0.420	0.919	0.614	0.726
Average	0.617	0.959	0.626	0.757	0.049	0.417	0.087	0.504	0.522	0.422	0.919	0.617	0.728

Supplementary Table 2. Metrics of classification performance of the PCA-SVM model for S1.
A, Performance of the PCA-SVM model using anti-GAD67, anti-NeuN, and Nissl signals in S1. ***B***, The same as ***A***, but using anti-NeuN and Nissl signals. ***C***, The same as ***A***, but using anti-GAD67 and Nissl signals. ***D***, The same as ***A***, but using anti-GAD67 and anti-NeuN signals. ***E***, The same as ***A***, but using an anti-GAD67 signal. ***F***, The same as ***A***, but using an anti-NeuN signal. ***G***, The same as ***A***, but using a Nissl signal. *Abbreviations*: S1, primary somatosensory cortex; PCA, principal component analysis; SVM, support vector machine.

A M1 FCN GAD67 + NeuN + Nissl

Cross validation	Accuracy	GAD67(-) precision	GAD67(-) recall	GAD67(-) F1 score	GAD67(+) precision	GAD67(+) recall	GAD67(+) F1 score	Macro precision	Macro recall	Macro F1 score	Weighted precision	Weighted recall	Weighted F1 score
1	0.898	0.998	0.894	0.943	0.338	0.968	0.502	0.668	0.931	0.722	0.963	0.898	0.920
2	0.915	0.998	0.912	0.953	0.381	0.963	0.546	0.690	0.938	0.750	0.965	0.915	0.932
3	0.921	0.997	0.920	0.957	0.398	0.946	0.560	0.697	0.933	0.759	0.965	0.921	0.936
4	0.873	0.998	0.867	0.928	0.292	0.976	0.449	0.645	0.921	0.689	0.961	0.873	0.903
5	0.930	0.996	0.929	0.962	0.426	0.934	0.585	0.711	0.932	0.773	0.966	0.930	0.942
Average	0.907	0.997	0.905	0.949	0.367	0.957	0.529	0.682	0.931	0.739	0.964	0.907	0.926

B M1 FCN NeuN + Nissl

Cross validation	Accuracy	GAD67(-) precision	GAD67(-) recall	GAD67(-) F1 score	GAD67(+) precision	GAD67(+) recall	GAD67(+) F1 score	Macro precision	Macro recall	Macro F1 score	Weighted precision	Weighted recall	Weighted F1 score
1	0.645	0.988	0.633	0.772	0.117	0.868	0.206	0.553	0.750	0.489	0.942	0.645	0.742
2	0.786	0.987	0.784	0.874	0.174	0.809	0.286	0.580	0.797	0.580	0.943	0.786	0.843
3	0.839	0.985	0.843	0.908	0.215	0.765	0.335	0.600	0.804	0.622	0.944	0.839	0.878
4	0.812	0.985	0.814	0.891	0.190	0.778	0.305	0.587	0.796	0.598	0.943	0.812	0.860
5	0.809	0.985	0.811	0.889	0.187	0.778	0.302	0.586	0.794	0.595	0.942	0.809	0.858
Average	0.778	0.986	0.777	0.867	0.176	0.800	0.287	0.581	0.788	0.577	0.943	0.778	0.836

C M1 FCN GAD67 + Nissl

Cross validation	Accuracy	GAD67(-) precision	GAD67(-) recall	GAD67(-) F1 score	GAD67(+) precision	GAD67(+) recall	GAD67(+) F1 score	Macro precision	Macro recall	Macro F1 score	Weighted precision	Weighted recall	Weighted F1 score
1	0.952	0.991	0.958	0.974	0.532	0.839	0.651	0.761	0.899	0.813	0.966	0.952	0.957
2	0.848	0.997	0.842	0.913	0.254	0.959	0.401	0.626	0.900	0.657	0.958	0.848	0.886
3	0.934	0.995	0.935	0.964	0.443	0.912	0.596	0.719	0.924	0.780	0.965	0.934	0.945
4	0.930	0.996	0.930	0.962	0.428	0.939	0.588	0.712	0.934	0.775	0.966	0.930	0.942
5	0.916	0.995	0.916	0.954	0.382	0.920	0.539	0.688	0.918	0.747	0.962	0.916	0.932
Average	0.916	0.995	0.916	0.953	0.408	0.914	0.555	0.701	0.915	0.754	0.964	0.916	0.932

D M1 FCN GAD67 + NeuN

Cross validation	Accuracy	GAD67(-) precision	GAD67(-) recall	GAD67(-) F1 score	GAD67(+) precision	GAD67(+) recall	GAD67(+) F1 score	Macro precision	Macro recall	Macro F1 score	Weighted precision	Weighted recall	Weighted F1 score
1	0.925	0.994	0.927	0.959	0.409	0.905	0.564	0.702	0.916	0.762	0.963	0.925	0.938
2	0.876	0.998	0.871	0.930	0.296	0.966	0.453	0.647	0.918	0.691	0.960	0.876	0.904
3	0.931	0.997	0.930	0.962	0.433	0.951	0.595	0.715	0.941	0.779	0.967	0.931	0.943
4	0.921	0.996	0.921	0.957	0.396	0.927	0.555	0.696	0.924	0.756	0.964	0.921	0.935
5	0.818	0.998	0.809	0.894	0.223	0.973	0.362	0.610	0.891	0.628	0.957	0.818	0.865
Average	0.894	0.997	0.891	0.940	0.351	0.944	0.506	0.674	0.918	0.723	0.962	0.894	0.917

Supplementary Table 3. Metrics of classification performance of the FCN-based model for M1 (continued on the next page).

E M1 FCN GAD67

Cross validation	Accuracy	GAD67(-) precision	GAD67(-) recall	GAD67(-) F1 score	GAD67(+) precision	GAD67(+) recall	GAD67(+) F1 score	Macro precision	Macro recall	Macro F1 score	Weighted precision	Weighted recall	Weighted F1 score
1	0.933	0.990	0.938	0.963	0.430	0.839	0.569	0.710	0.888	0.766	0.961	0.933	0.942
2	0.922	0.992	0.925	0.957	0.393	0.870	0.542	0.693	0.898	0.750	0.960	0.922	0.935
3	0.907	0.993	0.908	0.949	0.351	0.892	0.504	0.672	0.900	0.726	0.959	0.907	0.925
4	0.900	0.994	0.900	0.945	0.335	0.897	0.488	0.664	0.899	0.716	0.959	0.900	0.920
5	0.907	0.994	0.908	0.949	0.354	0.902	0.508	0.674	0.905	0.729	0.960	0.907	0.925
Average	0.914	0.993	0.916	0.953	0.373	0.880	0.522	0.683	0.898	0.737	0.960	0.914	0.930

F M1 FCN NeuN

Cross validation	Accuracy	GAD67(-) precision	GAD67(-) recall	GAD67(-) F1 score	GAD67(+) precision	GAD67(+) recall	GAD67(+) F1 score	Macro precision	Macro recall	Macro F1 score	Weighted precision	Weighted recall	Weighted F1 score
1	0.581	0.976	0.572	0.721	0.090	0.753	0.160	0.533	0.662	0.441	0.929	0.581	0.691
2	0.700	0.977	0.699	0.815	0.118	0.713	0.202	0.548	0.706	0.509	0.932	0.700	0.782
3	0.713	0.974	0.716	0.825	0.116	0.659	0.197	0.545	0.688	0.511	0.928	0.713	0.792
4	0.521	0.978	0.505	0.666	0.084	0.803	0.152	0.531	0.654	0.409	0.931	0.521	0.639
5	0.670	0.975	0.669	0.794	0.105	0.692	0.182	0.540	0.681	0.488	0.929	0.670	0.761
Average	0.637	0.976	0.632	0.764	0.102	0.724	0.179	0.539	0.678	0.471	0.930	0.637	0.733

G M1 FCN Nissl

Cross validation	Accuracy	GAD67(-) precision	GAD67(-) recall	GAD67(-) F1 score	GAD67(+) precision	GAD67(+) recall	GAD67(+) F1 score	Macro precision	Macro recall	Macro F1 score	Weighted precision	Weighted recall	Weighted F1 score
1	0.583	0.975	0.574	0.723	0.089	0.735	0.158	0.532	0.655	0.441	0.927	0.583	0.693
2	0.643	0.977	0.639	0.772	0.102	0.729	0.178	0.539	0.684	0.475	0.930	0.643	0.741
3	0.646	0.977	0.642	0.774	0.102	0.729	0.179	0.540	0.685	0.477	0.930	0.646	0.743
4	0.322	0.983	0.289	0.447	0.067	0.912	0.125	0.525	0.600	0.286	0.935	0.322	0.430
5	0.707	0.973	0.710	0.821	0.111	0.645	0.190	0.542	0.677	0.505	0.927	0.707	0.787
Average	0.580	0.977	0.571	0.707	0.094	0.750	0.166	0.536	0.660	0.437	0.930	0.580	0.679

Supplementary Table 3. Metrics of classification performance of the FCN-based model for M1.
A, Performance of the FCN-based model using anti-GAD67, anti-NeuN, and Nissl signals in M1. ***B***, The same as ***A***, but using anti-NeuN and Nissl signals. ***C***, The same as ***A***, but using anti-GAD67 and Nissl signals. ***D***, The same as ***A***, but using anti-GAD67 and anti-NeuN signals. ***E***, The same as ***A***, but using an anti-GAD67 signal. ***F***, The same as ***A***, but using an anti-NeuN signal. ***G***, The same as ***A***, but using a Nissl signal. *Abbreviations*: M1, primary motor cortex; FCN, fully convolutional network.

A M1 SVM GAD67 + NeuN + Nissl

Cross validation	Accuracy	GAD67(-) precision	GAD67(-) recall	GAD67(-) F1 score	GAD67(+) precision	GAD67(+) recall	GAD67(+) F1 score	Macro precision	Macro recall	Macro F1 score	Weighted precision	Weighted recall	Weighted F1 score
1	0.476	0.951	0.471	0.630	0.057	0.567	0.103	0.504	0.519	0.366	0.903	0.476	0.602
2	0.513	0.952	0.511	0.665	0.058	0.540	0.105	0.505	0.526	0.385	0.905	0.513	0.635
3	0.498	0.951	0.495	0.651	0.057	0.548	0.104	0.504	0.521	0.377	0.904	0.498	0.622
4	0.518	0.953	0.517	0.670	0.059	0.540	0.107	0.506	0.529	0.388	0.905	0.518	0.640
5	0.556	0.952	0.559	0.705	0.059	0.496	0.106	0.506	0.528	0.405	0.905	0.556	0.673
Average	0.512	0.952	0.511	0.664	0.058	0.538	0.105	0.505	0.525	0.385	0.904	0.512	0.634

B M1 SVM NeuN + Nissl

Cross validation	Accuracy	GAD67(-) precision	GAD67(-) recall	GAD67(-) F1 score	GAD67(+) precision	GAD67(+) recall	GAD67(+) F1 score	Macro precision	Macro recall	Macro F1 score	Weighted precision	Weighted recall	Weighted F1 score
1	0.456	0.949	0.449	0.610	0.055	0.570	0.100	0.502	0.510	0.355	0.902	0.456	0.583
2	0.497	0.952	0.494	0.651	0.058	0.555	0.105	0.505	0.525	0.378	0.904	0.497	0.622
3	0.504	0.953	0.502	0.657	0.059	0.555	0.106	0.506	0.528	0.382	0.905	0.504	0.628
4	0.512	0.951	0.510	0.664	0.058	0.533	0.104	0.504	0.522	0.384	0.904	0.512	0.635
5	0.551	0.952	0.554	0.700	0.060	0.506	0.107	0.506	0.530	0.404	0.905	0.551	0.669
Average	0.504	0.951	0.502	0.656	0.058	0.544	0.104	0.505	0.523	0.380	0.904	0.504	0.627

C M1 SVM GAD67 + Nissl

Cross validation	Accuracy	GAD67(-) precision	GAD67(-) recall	GAD67(-) F1 score	GAD67(+) precision	GAD67(+) recall	GAD67(+) F1 score	Macro precision	Macro recall	Macro F1 score	Weighted precision	Weighted recall	Weighted F1 score
1	0.496	0.950	0.493	0.649	0.056	0.538	0.102	0.503	0.516	0.376	0.903	0.496	0.620
2	0.556	0.951	0.560	0.705	0.059	0.489	0.105	0.505	0.524	0.405	0.904	0.556	0.673
3	0.542	0.950	0.545	0.692	0.057	0.489	0.102	0.503	0.517	0.397	0.903	0.542	0.661
4	0.542	0.950	0.545	0.692	0.057	0.487	0.101	0.503	0.516	0.397	0.902	0.542	0.661
5	0.597	0.950	0.606	0.740	0.058	0.435	0.103	0.504	0.521	0.422	0.903	0.597	0.706
Average	0.546	0.950	0.550	0.696	0.057	0.488	0.103	0.504	0.519	0.399	0.903	0.546	0.664

D M1 SVM GAD67 + NeuN

Cross validation	Accuracy	GAD67(-) precision	GAD67(-) recall	GAD67(-) F1 score	GAD67(+) precision	GAD67(+) recall	GAD67(+) F1 score	Macro precision	Macro recall	Macro F1 score	Weighted precision	Weighted recall	Weighted F1 score
1	0.515	0.951	0.514	0.667	0.058	0.533	0.105	0.505	0.523	0.386	0.904	0.515	0.637
2	0.659	0.950	0.676	0.790	0.060	0.369	0.103	0.505	0.522	0.446	0.903	0.659	0.753
3	0.563	0.952	0.567	0.711	0.060	0.489	0.106	0.506	0.528	0.408	0.904	0.563	0.678
4	0.603	0.949	0.613	0.745	0.057	0.418	0.101	0.503	0.516	0.423	0.902	0.603	0.711
5	0.705	0.952	0.726	0.823	0.065	0.342	0.110	0.509	0.534	0.467	0.905	0.705	0.785
Average	0.609	0.951	0.619	0.747	0.060	0.430	0.105	0.505	0.525	0.426	0.904	0.609	0.713

Supplementary Table 4. Metrics of classification performance of the PCA-SVM model for M1 (continued on the next page).

E M1 SVM GAD67

Cross validation	Accuracy	GAD67(-) precision	GAD67(-) recall	GAD67(-) F1 score	GAD67(+) precision	GAD67(+) recall	GAD67(+) F1 score	Macro precision	Macro recall	Macro F1 score	Weighted precision	Weighted recall	Weighted F1 score
1	0.501	0.951	0.499	0.654	0.057	0.543	0.104	0.504	0.521	0.379	0.904	0.501	0.625
2	0.743	0.950	0.769	0.850	0.064	0.281	0.104	0.507	0.525	0.477	0.903	0.743	0.811
3	0.719	0.949	0.742	0.833	0.061	0.296	0.100	0.505	0.519	0.467	0.902	0.719	0.794
4	0.671	0.948	0.690	0.799	0.056	0.328	0.096	0.502	0.509	0.447	0.901	0.671	0.761
5	0.781	0.951	0.811	0.875	0.071	0.257	0.111	0.511	0.534	0.493	0.904	0.781	0.835
Average	0.683	0.950	0.702	0.802	0.062	0.341	0.103	0.506	0.522	0.453	0.903	0.683	0.765

F M1 SVM NeuN

Cross validation	Accuracy	GAD67(-) precision	GAD67(-) recall	GAD67(-) F1 score	GAD67(+) precision	GAD67(+) recall	GAD67(+) F1 score	Macro precision	Macro recall	Macro F1 score	Weighted precision	Weighted recall	Weighted F1 score
1	0.497	0.950	0.495	0.651	0.057	0.540	0.103	0.504	0.518	0.377	0.903	0.497	0.622
2	0.467	0.953	0.459	0.620	0.058	0.597	0.106	0.506	0.528	0.363	0.906	0.467	0.593
3	0.478	0.951	0.473	0.631	0.056	0.562	0.103	0.504	0.518	0.367	0.903	0.478	0.603
4	0.515	0.954	0.513	0.667	0.060	0.557	0.109	0.507	0.535	0.388	0.906	0.515	0.637
5	0.610	0.953	0.618	0.750	0.062	0.452	0.110	0.508	0.535	0.430	0.905	0.610	0.716
Average	0.513	0.952	0.512	0.664	0.059	0.542	0.106	0.505	0.527	0.385	0.905	0.513	0.634

G M1 SVM Nissl

Cross validation	Accuracy	GAD67(-) precision	GAD67(-) recall	GAD67(-) F1 score	GAD67(+) precision	GAD67(+) recall	GAD67(+) F1 score	Macro precision	Macro recall	Macro F1 score	Weighted precision	Weighted recall	Weighted F1 score
1	0.455	0.950	0.449	0.609	0.055	0.575	0.101	0.502	0.512	0.355	0.902	0.455	0.582
2	0.516	0.952	0.515	0.668	0.058	0.533	0.105	0.505	0.524	0.387	0.904	0.516	0.638
3	0.540	0.951	0.542	0.691	0.058	0.499	0.103	0.504	0.520	0.397	0.903	0.540	0.659
4	0.506	0.951	0.504	0.659	0.057	0.533	0.103	0.504	0.519	0.381	0.903	0.506	0.630
5	0.478	0.950	0.474	0.632	0.056	0.560	0.102	0.503	0.517	0.367	0.903	0.478	0.604
Average	0.499	0.951	0.497	0.652	0.057	0.540	0.103	0.504	0.518	0.377	0.903	0.499	0.623

Supplementary Table 4. Metrics of classification performance of the PCA-SVM model for M1.

A, Performance of the PCA-SVM model using anti-GAD67, anti-NeuN, and Nissl signals in M1. ***B***, The same as ***A***, but using anti-NeuN and Nissl signals. ***C***, The same as ***A***, but using anti-GAD67 and Nissl signals. ***D***, The same as ***A***, but using anti-GAD67 and anti-NeuN signals. ***E***, The same as ***A***, but using an anti-GAD67 signal. ***F***, The same as ***A***, but using an anti-NeuN signal. ***G***, The same as ***A***, but using a Nissl signal. *Abbreviations*: M1, primary motor cortex; PCA, principal component analysis; SVM, support vector machine.

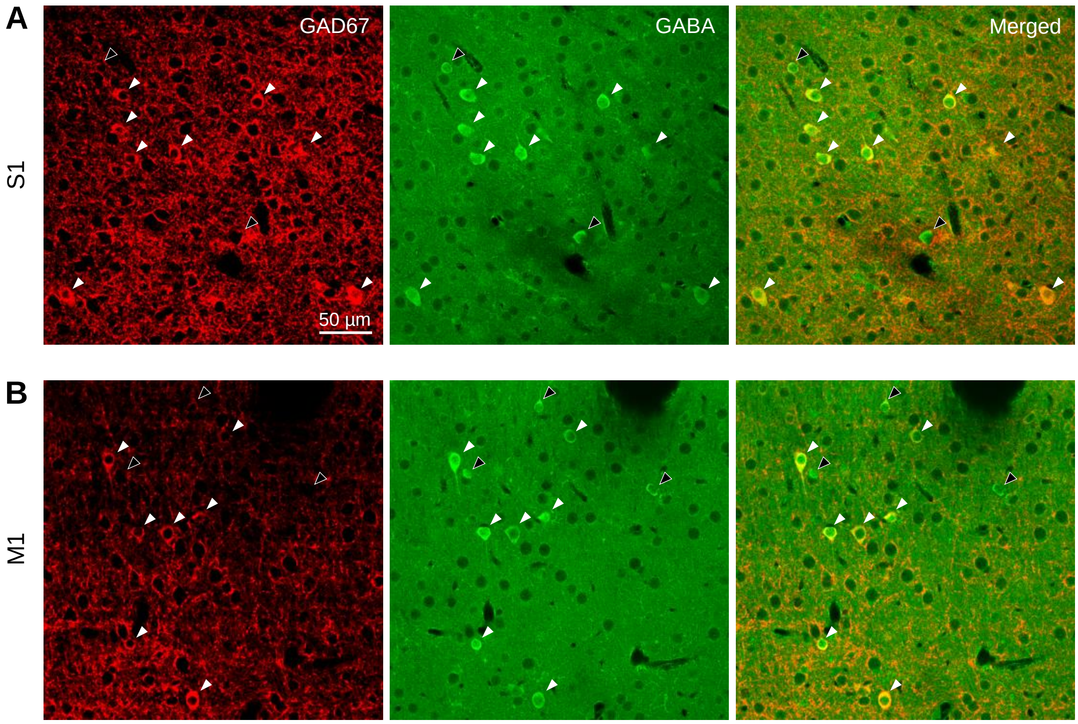

Supplementary Figure 1. Representative photographs of GAD67 and GABA immunoreactivity in S1 and M1. ***A***, Representative image (2048 × 2048 pixels, 16-bit intensity, 40×) of a section of S1 immunostained for GAD67 (*red*, *leftmost* (*first*)) and GABA (*green*, *second*). A merged image is shown in the *third* panel. Cells double-immunopositive for GAD67 and GABA are indicated by *white* arrowheads, whereas GABA-immunopositive (but GAD67-immunonegative) cells are indicated by *black* arrowheads. ***B***, The same as ***A***, but for M1. Note that we used rabbit primary antibody against GABA (1:500, A2052, Merck, NJ, USA) for GABA-immunostaining. *Abbreviations*: S1, primary somatosensory cortex; M1, primary motor cortex.

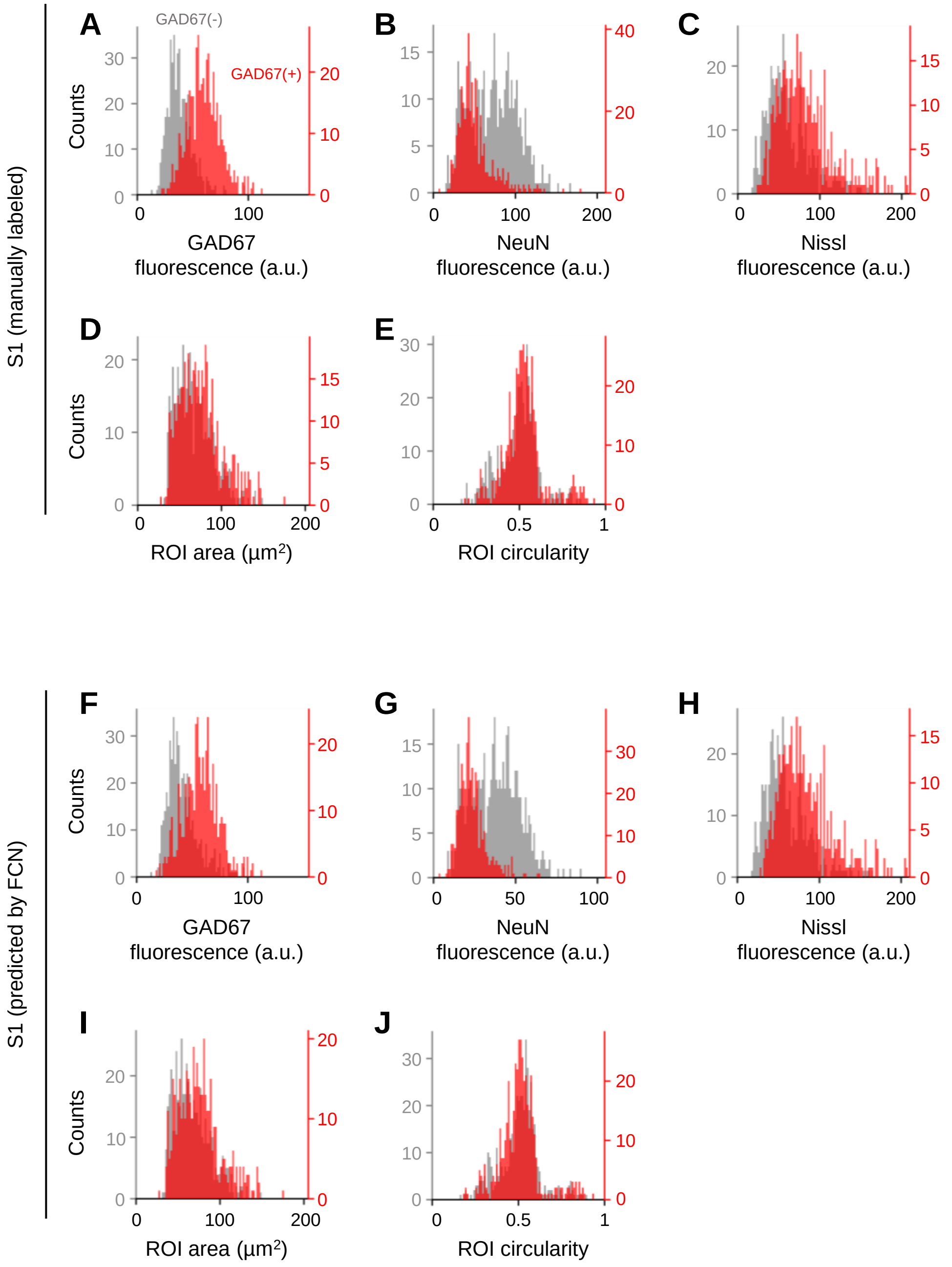

Supplementary Figure 2. Differences in the immunofluorescence and shape of GAD67-positive and GAD67-negative cells in S1 manually as annotated or predicted by deep learning. ***A***, Histogram of the fluorescence of anti-GAD67 signals from manually annotated GAD67-positive (*red*) and -negative (*gray*) neurons in S1. ***B***, The same as ***A***, but for anti-NeuN immunofluorescence signals. ***C***, The same as ***A***, but for Nissl fluorescence signals. ***D***, The same as ***A***, but for the area of individual neurons (*i.e.*, ROIs). ***E***, The same as ***A***, but for the circularity of individual neurons (*i.e.*, ROIs). ***F–J***, The same as ***A–E***, respectively, but for the cell images predicted by the FCN model pretrained on NeuN and Nissl fluorescence. *Abbreviations*: S1, primary somatosensory cortex; FCN, fully convolutional network; ROI, region of interest.

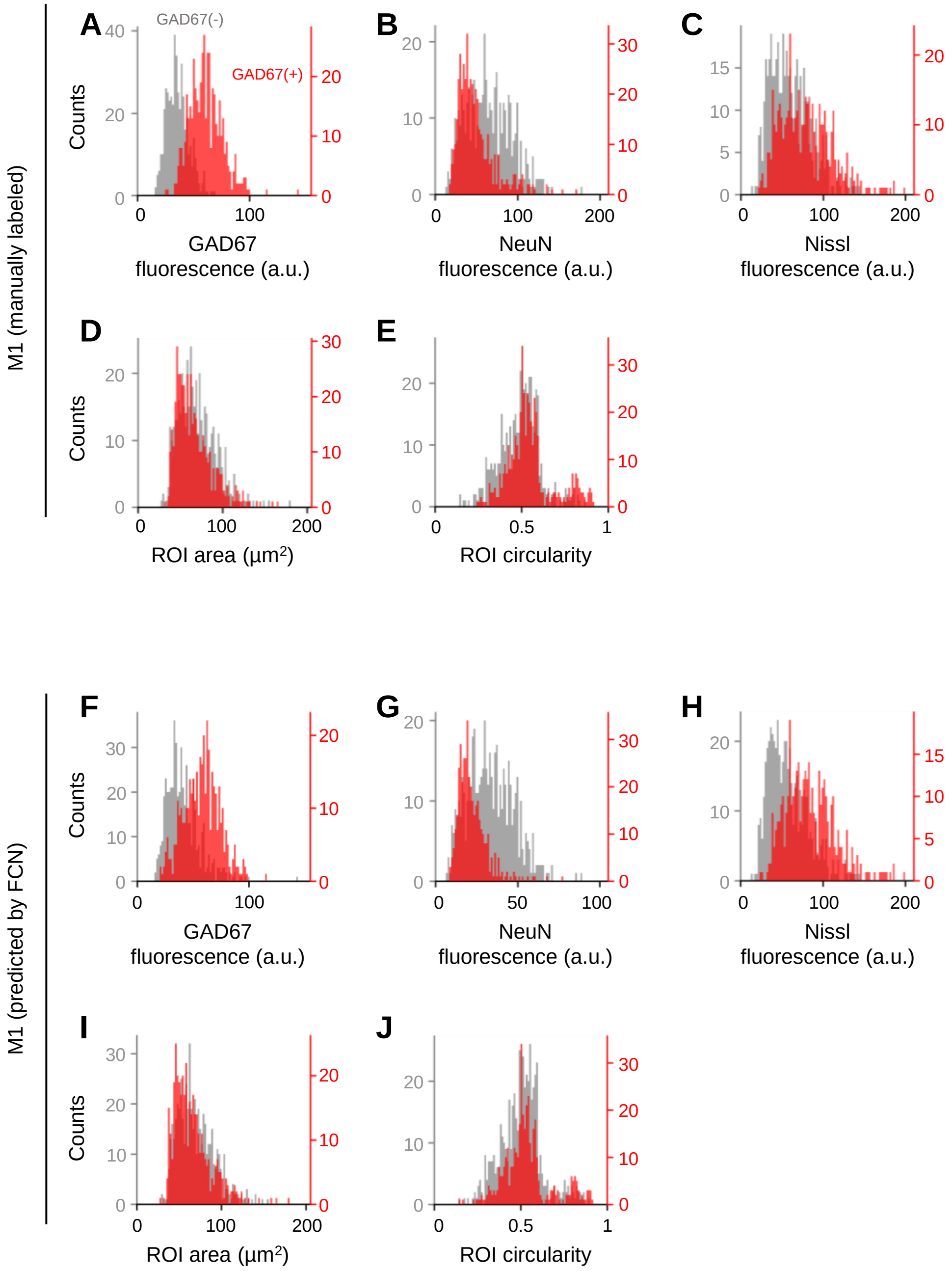

Supplementary Figure 3. Differences in the immunofluorescence and shape of GAD67-positive and GAD67-negative cells in M1 as manually annotated or predicted by deep learning. ***A***, Histogram of the fluorescence of anti-GAD67 signals from manually annotated GAD67-positive (*red*) and -negative (*gray*) neurons in M1. ***B***, The same as ***A***, but for anti-NeuN immunofluorescence signals. ***C***, The same as ***A***, but for Nissl fluorescence signals. ***D***, The same as ***A***, but for the area of individual neurons (*i.e.*, ROIs). ***E***, The same as ***A***, but for the circularity of individual neurons (*i.e.*, ROIs). ***F–J***, The same as ***A–E***, respectively, but for the cell images predicted by the FCN model pretrained on NeuN and Nissl fluorescence. *Abbreviations*: M1, primary motor cortex; FCN, fully convolutional network; ROI, region of interest.

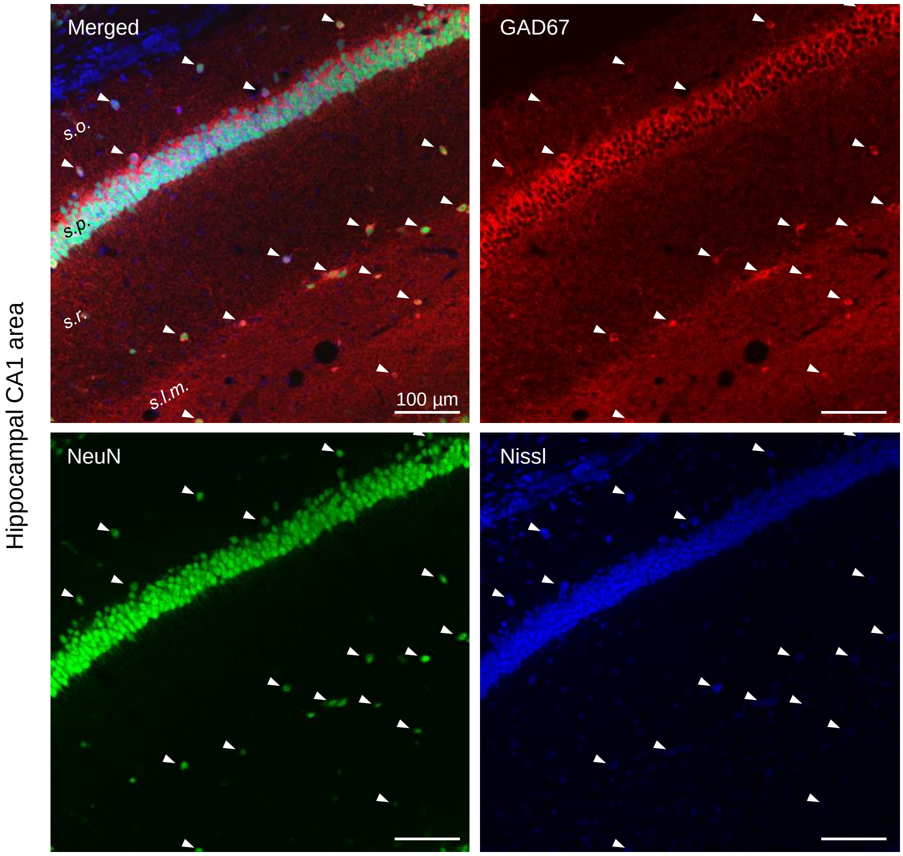

Supplementary Figure 4. Representative photographs of GAD67 and NeuN immunoreactivity and Nissl reactivity in the hippocampal CA1 area. Representative fluorescence image (1024 × 1024 pixels, 16-bit intensity, 20×) of the hippocampal CA1 area. These sections were immunostained for GAD67 (*top right* (*red*)) and NeuN (*bottom left* (*green*)) and stained for Nissl substances (*bottom right* (*blue*)). A merged image is displayed in the *top left* panel. GAD67-immunopositive cells are indicated by *white* arrowheads. *Abbreviations*: *s.o., stratum oriens*; *s.p., stratum pyramidale*; *s.r., stratum radiatum*; *s.l.m., stratum lacunosum moleculare*.

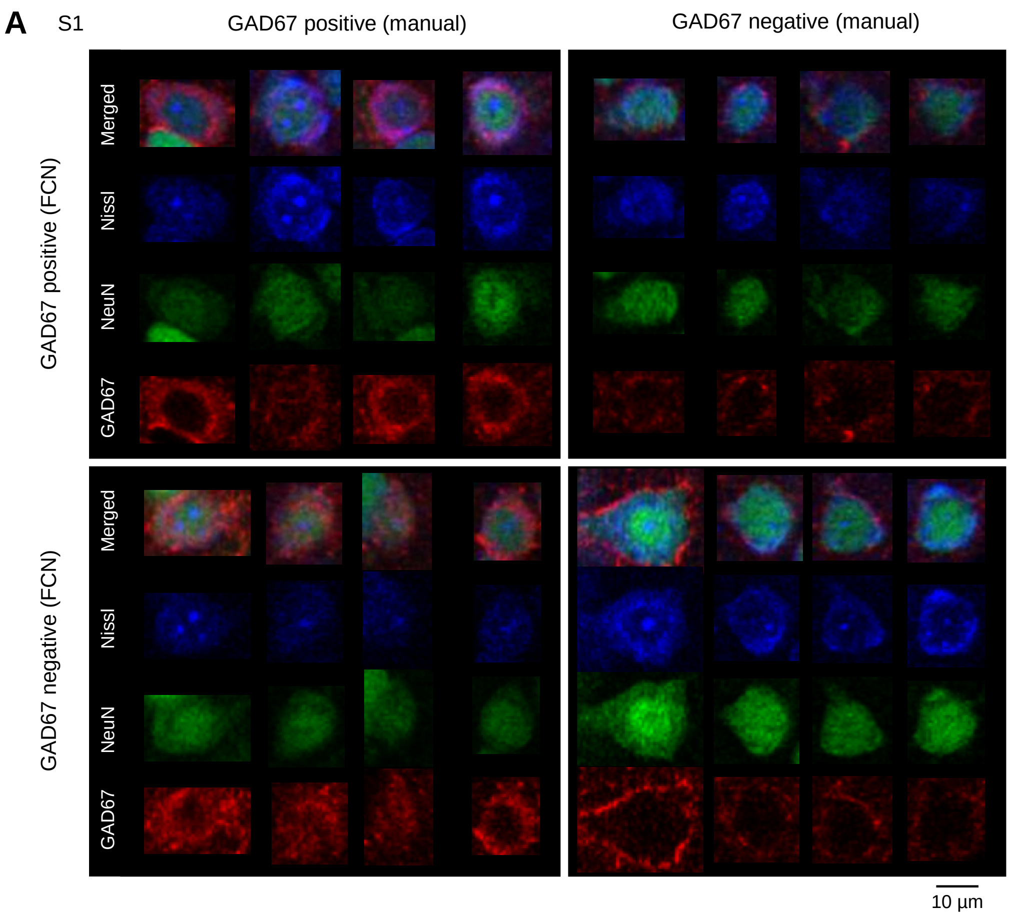

Supplementary Figure 5. Representative photographs of GAD67 and NeuN immunoreactivity and Nissl reactivity in single neocortical cells categorized by experimenters and deep learning. ***A***, *Top left*: Representative fluorescence images of four S1 neurons that were manually labeled as positive and predicted as positive by the FCN model pretrained on NeuN-immunofluorescence and Nissl-fluorescence images. The cells were immunostained for GAD67 (*red*), NeuN (*green*), and Nissl (*blue*). *Top right*: The same as *top left*, but for cell images that were manually annotated as negative but predicted as positive by the FCN model. *Bottom left*: The same as *top left*, but for cell images that were manually annotated as positive but predicted as negative by the FCN model. *Bottom right*: The same as *top left*, but for cell images that were manually annotated as negative and predicted as negative by the FCN model. *Abbreviations*: S1, primary somatosensory cortex; FCN, fully convolutional network.

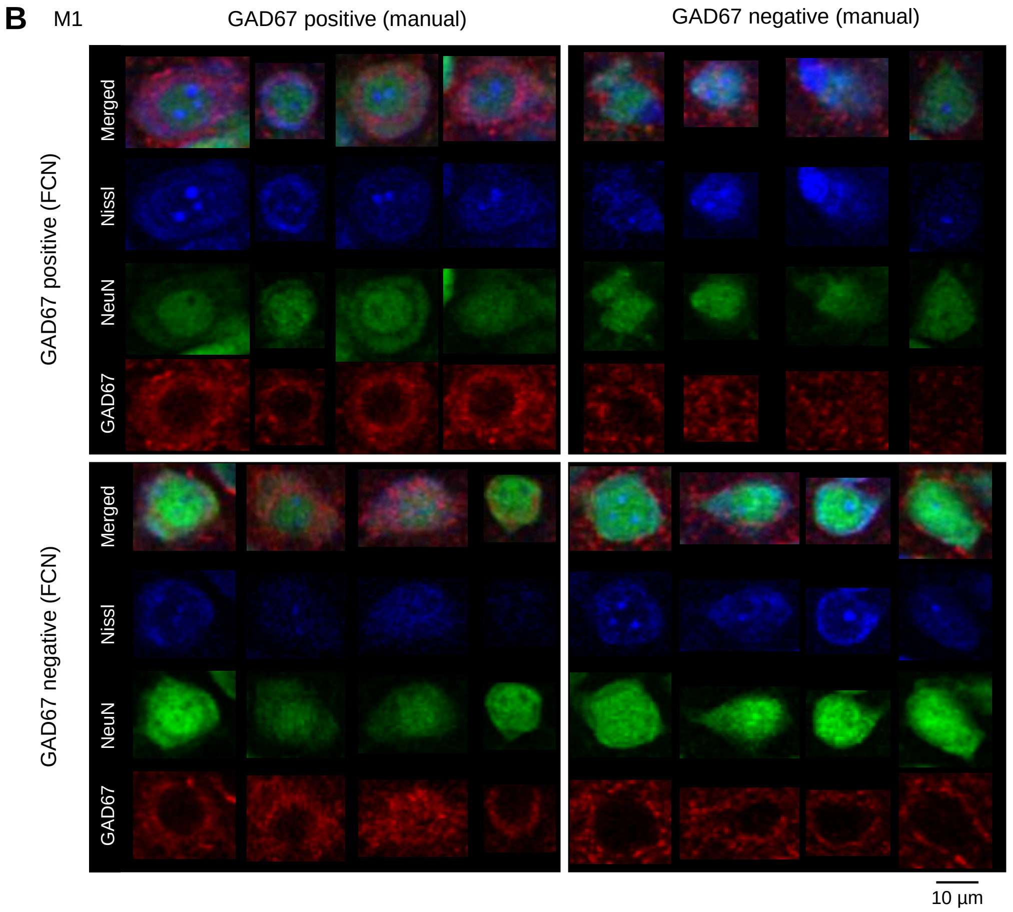

Supplementary Figure 5. Representative photographs of GAD67 and NeuN immunoreactivity and Nissl reactivity in single neocortical cells categorized by experimenters and deep learning (continued from the previous page). ***B***, The same as ***A***, but for M1 neurons. *Abbreviations*: M1, primary motor cortex; FCN, fully convolutional network.
